# Charged organic ligands inserting/supporting the nanolayer spacing of vanadium oxides for high-stability/efficiency zinc-ion batteries

**DOI:** 10.1093/nsr/nwae336

**Published:** 2024-09-20

**Authors:** Guoqiang Yuan, Yichun Su, Xiangling Zhang, Biao Gao, Jinliang Hu, Yangyang Sun, Wenting Li, Zhan Zhang, Mohsen Shakouri, Huan Pang

**Affiliations:** School of Chemistry and Chemical Engineering, Yangzhou University, Yangzhou 225009, China; School of Chemistry and Chemical Engineering, Yangzhou University, Yangzhou 225009, China; School of Chemistry and Chemical Engineering, Yangzhou University, Yangzhou 225009, China; School of Chemistry and Chemical Engineering, Yangzhou University, Yangzhou 225009, China; Jiangsu Yangnong Chemical Group Co. Ltd., Yangzhou 225009, China; School of Chemistry and Chemical Engineering, Yangzhou University, Yangzhou 225009, China; School of Chemistry and Chemical Engineering, Yangzhou University, Yangzhou 225009, China; School of Chemistry and Chemical Engineering, Yangzhou University, Yangzhou 225009, China; Canadian Light Source Inc., University of Saskatchewan, Saskatoon S7N 2V3, Canada; School of Chemistry and Chemical Engineering, Yangzhou University, Yangzhou 225009, China

**Keywords:** coordination chemistry, organic carboxylic acid ligand, ion migration, layered vanadium oxide, aqueous zinc-ion battery

## Abstract

Given their high safety, environmental friendliness and low cost, aqueous zinc-ion batteries (AZIBs) have the potential for high-performance energy storage. However, issues with the structural stability and electrochemical kinetics during discharge/charge limit the development of AZIBs. In this study, vanadium oxide electrodes with organic molecular intercalation were designed based on intercalating 11 kinds of charged organic carboxylic acid ligands between 2D layers to regulate the interlayer spacing. The negatively charged carboxylic acid group can neutralize Zn^2+^, reduce electrostatic repulsion and enhance electrochemical kinetics. The intercalated organic molecules increased the interlayer spacing. Among them, the 0.028EDTA · 0.28NH_4_^+^ · V_2_O_5_ · 0.069H_2_O was employed as the cathode with a high specific capacity (464.6 mAh g^−1^ at 0.5 A g^−1^) and excellent rate performance (324.4 mAh g^−1^ at 10 A g^−1^). Even at a current density of 20 A g^−1^, the specific capacity after 2000 charge/discharge cycles was 215.2 mAh g^−1^ (capacity retention of 78%). The results of this study demonstrate that modulation of the electrostatic repulsion and interlayer spacing through the intercalation of organic ligands can enhance the properties of vanadium-based materials.

## Teaser

This work demonstrates the intercalation of charged organic carboxylic acids into the interlayer structure of vanadium oxides, constructing stable ion transport channels, and achieving efficient aqueous zinc-ion battery energy storage.

## INTRODUCTION

Aqueous zinc-ion batteries (AZIBs) have emerged as a promising energy-storage device owing to their high safety, low cost, high theoretical specific capacity (820 mAh g^−1^) and low redox potential [1–4]. Recently, various types of manganese oxides, vanadium oxides (VO) and Prussian blue analogs have been used as cathodes for AZIBs [5–10]. Among these, VO have attracted significant research interest because of their multivalent state and complex crystal structure [11–13]. However, 2D layered VO still suffer from small nanolayer spacing, easy structural collapse and poor ionic conductivity. In addition, the large electrostatic repulsion and high hydration between the Zn^2+^ during discharge/charge lead to slow electrochemical kinetics [14,15]. Stable and precise regulation of the VO interlayer spacing is considered an effective means of accomplishing efficient energy storage in AZIBs [16,17].

Intercalation is a practical method for precisely controlling the spacing between VO nanolayers [18–20]. Layered VO have open 2D channels that provide spatial sites for intercalated species, such as water molecules, metal ions, ammonium ions and organic molecules, as well as some of their co-intercalated layers. For example, the insertion of metal ions, such as Li^+^, Na^+^, K^+^, Zn^2+^ and Al^3+^, expands the interlayer spacing of the VO structures [21–25]. However, the electrochemical stability of VO remains poor owing to the structural instability caused by changes in the insertion and volume of metal ions [26–28]. Organic molecular intercalation increases the interlayer spacing, thereby accelerating the diffusion kinetics. Additionally, abundant functional groups are provided outside the active sites. For example, polyvinylpyrrolidone [5], poly(3,4-ethylenedioxythiophene) [29], ethylenediamine [30] and 1,3-propylenediamine [12] improve the nanolayer spacing when inserted into layered oxides and achieve stable cycling performance.

**Figure 1.**
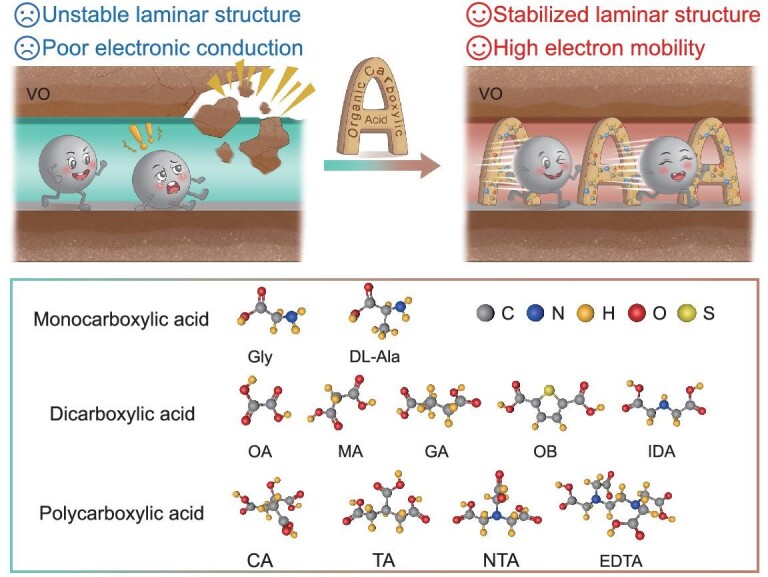
Schematic representation of Zn^2+^ migration in vanadium oxides (VO) intercalated with three types of organic carboxylic acids: monocarboxylic acids, dicarboxylic acids and polycarboxylic acids. Glycine (Gly), DL-alanine (DL-Ala), oxalic acid (OA), malonic acid (MA), glutaric acid (GA), 2,5-thiophenedicarboxylic acid (OB), iminodiacetic acid (IDA), 1,2,3-propanetricarboxylic acid (TA), citric acid (CA), nitrilotriacetic acid (NTA), ethylenediaminetetraacetic acid (EDTA).

However, the role of charged organic ligands in inserting/supporting the nanolayer spacing of VO, which plays an important role in the synthesis of metal–organic frameworks, has rarely been investigated. In this study, charged organic carboxylic acid ligands were utilized as structural scaffolds to construct stable ion transport channels in VO nanolayers to significantly enhance the electrochemical properties of AZIBs (Fig. 1). Meanwhile, the electrostatic interactions of Zn^2+^ were reduced by negatively charged organic soluble acid molecules (Fig. S1). The improvement in the VO layer spacing and change in the valence of V induced by different organic carboxylic acid ligands were systematically investigated. The effect of organic carboxylic acid intercalation on the valence of V was analysed by using X-ray absorption fine structure spectroscopy. In addition, *ex situ* and *in situ* tests and galvanostatic intermittent titration technique (GITT) calculations demonstrated that organic carboxylic acids have a significant function in stabilizing the laminar structure, inhibiting vanadium solvation and facilitating ion migration during discharge/charge. This study reveals the effects of changes in the valence of V and interlayer spacing on the structural stability and electrochemical properties VO that are intercalated with charged organic carboxylic acid ligands, providing insights for the design of excellent cathode materials suitable for AZIBs.

## RESULTS AND DISCUSSION

**Figure 2.**
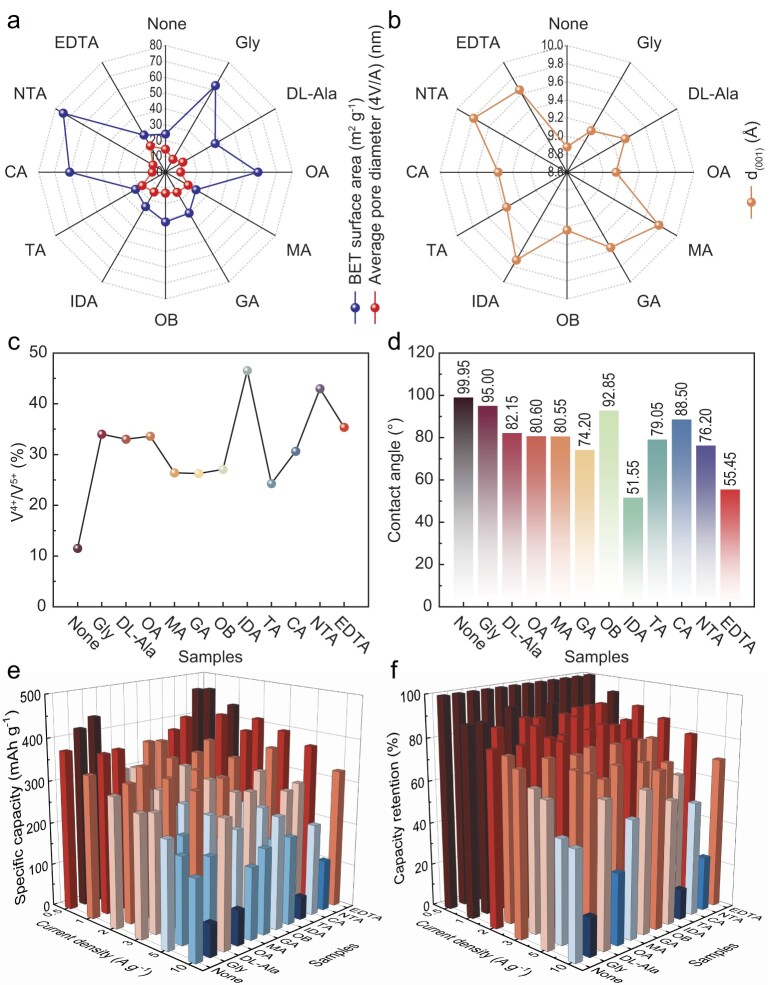
Comparative radar diagram of (a) BET surface area, average pore diameter and (b) interlayer spacing for different materials. (c) The ratio of V^4+^/V^5+^. (d) The contact angle measurement of the electrode with the electrolyte. Histogram of (e) specific capacity and (f) capacity retention of different samples at different current densities.

Charged organic carboxylic acids intercalate into the layered VO by inserting molecules of these acids between the 2D VO layers by using a hydrothermal method. The organic carboxylic acids studied are shown in Fig. 1. The prepared materials were designated as None, Gly (glycine), DL-Ala (DL-Alanine), OA (oxalic acid), MA (malonic acid), GA (glutaric acid), OB (thiophenedicarboxylic acid), IDA (iminodiacetic acid), TA (1,2,3-(propanetricarboxylic acid)), CA (citric acid), NTA (nitrilotriacetic acid) and EDTA (ethylenediaminetetraacetic acid), according to the corresponding organic carboxylic acids used. The morphologies of all the products were determined to be nanoribbon structures by using scanning electron microscopy (SEM) and transmission electron microscopy (TEM) (Figs S2 and S3). However, the nanobelt lengths of the different intercalated organic carboxylic acids varied. Notably, the EDTA exhibited highly consistent structural dimensions, which were particularly prominent in the samples. The X-ray diffraction (XRD) patterns and Raman spectrum demonstrated that the organic carboxylic acid intercalation did not modify the physical phase or crystal structure of the VO (Figs S4 and S5). The determination of the elements C, H and N based on the C, H, N and S elemental analysis test showed the presence of organic carboxylic acids in materials (Table S1). The presence of organic carboxylic acids was validated by using Fourier transform infrared (FTIR) spectra (Fig. S6). The characteristic peak at 1647 cm^−1^ is attributed to the stretching vibration of –COOH in the organic carboxylic acids [31,32]. The insertion of organic carboxylic acids led to an increase in the Brunauer–Emmett–Teller (BET) specific surface area and the average pore size of the nanoribbons (Fig. 2a, Fig. S7 and Table S2). Moreover, there was a multistage pore advantage that facilitated the intercalation and deintercalation of Zn^2+^ (Fig. S8). The high-resolution TEM (HRTEM) images demonstrate that the inclusion of organic carboxylic acids caused an expansion in the lattice spacing of the samples (Fig. 2b and Fig. S9). Simultaneously, the lattice spacing generally showed a regular increase with an increasing number of molecular chains. The clearly defined lattice diffraction fringes indicate that the material had excellent crystallinity. According to the energy-dispersive X-ray (EDX) elemental mapping images, the distribution of C, N, O and V on the nanoribbons was uniform (Figs S9 and S10). As shown in Fig. S11, the mass loss is the removal of adsorbed water until 150°C. The mass change between 150°C and 500°C is due to the loss of structural water, NH_4_^+^ and organic carboxylic acids. The X-ray photoelectron spectroscopy (XPS) spectrum of V 2p shows characteristic peaks at ∼517.5 and ∼516.1 eV, corresponding to V^5+^ and V^4+^, respectively (Fig. S12) [33]. In addition, the inclusion of organic carboxylic acids led to a partial reduction in V^5+^ and an increase in V^4+^ (Fig. 2c). This suggests that the presence of organic carboxylic acids changes not only the interlayer spacing, but also the valence of V.

Relevant electrochemical tests were conducted on all materials to investigate the effect of different organic carboxylic acid embeddings on the electrochemical properties of the VO. Through testing the contact angles of the electrodes, it was found that the insertion of organic carboxylic acids reduced the contact angle between the electrolyte and the electrode, thereby enhancing the hydrophilicity of the electrode (Fig. 2d and Fig. S13). This facilitates better electrolyte wetting of the electrode, enhances ion transport and reduces impedance (Fig. S14). Notably, the EDTA electrode stands out in this aspect. The electrode cyclic voltammetry (CV) curves in the range of 0.2–1.0 mV s^−1^ are presented in Fig. S15. Each CV curve shows two pairs of redox peaks. However, as will be discussed later, the production of Zn–N during discharge/charge caused the peaks of IDA, NTA and EDTA at the voltage interval of 1.3118/1.3015 V. The CV curves show considerable overlap at different numbers of turns at 1.0 mV s^−1^, demonstrating that the electrode materials had outstanding cyclic stability (Fig. S16). The rate performance of the electrode materials is shown in Fig. S17. The cell capacity could still be returned to its starting point when the current density was raised from 0.5 to 10 A g^−1^ and back again, indicating excellent rate performance. Remarkably, the EDTA electrode had a higher specific capacity and capacity retention than the other electrodes (Fig. 2e and f). The electrode was submerged in the electrolyte for 30 days without substantial electrode dissolution, proving that the electrode material was highly stable in the electrolyte (Figs S18 and S19). The study indicates that, compared with other organic carboxylic acid-intercalated materials, EDTA-intercalated VO not only exhibit highly uniform morphology and size, increased interlayer spacing and stable interlayer structure, but also possess excellent conductivity and outstanding electrochemical performance.

To gain further insight into the influence of the organic carboxylic acid content on the structural and electrochemical properties of VO, we investigated VO that were intercalated with various concentrations of EDTA. The samples were named based on their EDTA content: EDTA-0.0 (0.0 mM EDTA), EDTA-1.0 (1.0 mM EDTA), EDTA-2.5 (2.5 mM EDTA) and EDTA-5.0 (5.0 mM EDTA). The determination of each element based on the C, H, N and S elemental analyses indicates different EDTA contents in the different materials (Table S3). Compared with the other samples, EDTA-1.0 exhibits a more homogeneous and stable morphological structure (Figs S20 and S21) and elemental distribution (Figs S22 and S23). Moreover, the BET test findings reveal that varied quantities of incorporated EDTA generated a proportionate increase in the specific surface area and pore size of the materials, with the highest pore size for EDTA-1.0 (Figs S24 and S25 and Table S3). As shown in Fig. S26a, the samples exhibited a monoclinic structure, which was consistent with the standard data (JCPDS no. 415–4512, V_2_O_5_·H_2_O). The diffraction peaks of the (001) crystal face become sharper, which indicates that the EDTA in the sample affected the crystallinity (Fig. S26a). Moreover, the (001) crystal plane is left-shifted to different degrees, indicating that the intercalation of the EDTA increased the structural interlayer spacing (Fig. S26b). The peak at 1332 cm^−1^ is attributed to the stretching vibration of the C–N bond [31]. The peak intensity varies depending on the amount of EDTA present (Fig. S27). The mode at 159 cm^−1^ is indicative of the V–O–V–O bending vibration mode (Fig. S28) [34]. The blue shift at this position indicates the compressive deformation of the sample structure along the *a*-axis. This deformation is due to the electrostatic interactions between the organic carboxylic acid molecules and the V atoms. Additionally, XPS demonstrates that the intensity of the V^5+^ component was partially reduced due to the intercalation of the EDTA (Fig. S29). The chemical formulas of the various substances can be determined as 0.33NH_4_^+^ · V_2_O_5_ · 0.67H_2_O (EDTA-0.0), 0.028EDTA · 0.28NH_4_^+^ · V_2_O_5_ · 0.069H_2_O (EDTA-1.0), 0.013EDTA · 0.323NH_4_^+^ · V_2_O_5_ · 0.419H_2_O (EDTA-2.5) and 0.050EDTA · 0.182NH_4_^+^ · V_2_O_5_ · 0.002H_2_O (EDTA-5.0) based on the thermogravimetric results and C, H, N and S elemental analysis (Fig. S30 and Table S4).

**Figure 3.**
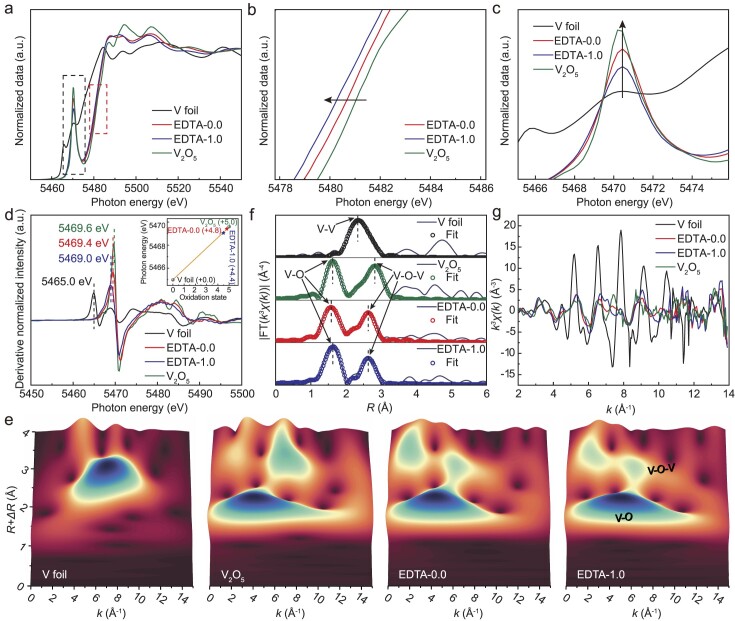
(a) V K-edge XANES spectra for V foil, V_2_O_5_, EDTA-0.0, and EDTA-1.0. (b) and (c) Enlarged XANES spectra from (a). (d) Corresponding first derivatives of XANES for V foil, V_2_O_5_, EDTA-0.0, and EDTA-1.0. (e) Wavelet transform plots for V foil, V_2_O_5_, EDTA-0.0, and EDTA-1.0. (f) Fitting of the magnitude of the Fourier transform of the EXAFS for V foil, V_2_O_5_, EDTA-0.0, and EDTA-1.0. (g) V K-edge EXAFS *k*^3^*–χ*(*k*) oscillation curves.

X-ray absorption spectroscopy was conducted to investigate the atomic arrangement and coordination environment, providing a detailed analysis of the atomic structure and valence. Figure 3a depicts local zoomed-in plots which indicate that the absorption edges of EDTA-0.0 and EDTA-1.0 are shifted towards lower energies compared with commercial V_2_O_5_. Notably, EDTA-1.0 shows the greatest shift (Fig. 3b). Analysis of the pre-edge peak reveals that the V_2_O_5_ peak was stronger than those of EDTA-0.0 and EDTA-1.0, indicating that V_2_O_5_ had a higher valence than EDTA-0.0 and EDTA-1.0 (Fig. 3c) [35]. The average valence information of EDTA-0.0 and EDTA-1.0 was obtained by using linear fitting, as shown in the inset of Fig. 3d, combined with the first-order derivatives of X-ray absorption near-edge structure (XANES). In EDTA-0.0 and EDTA-1.0, the average valence of V is +4.8 and +4.4, respectively [36]. These results suggest that EDTA regulates the chemical state of V, as confirmed by the XPS results. The Fourier transform and wavelet transform images of the extended X-ray absorption fine-structure spectroscopy (EXAFS) spectra of V demonstrate that EDTA-0.0, EDTA-1.0 and V_2_O_5_ exhibit comparable structural characteristics (Fig. 3e and Fig. S31). Moreover, the average integer coordination number of V–O in EDTA-0.0 and EDTA-1.0 is 5.0, which is identical to that of V_2_O_5_ (Table S5). In Fig. 3f, the EXAFS fitting parameter of the V K-edge indicates that there is no significant change in the V–O bond distances of EDTA-0.0 and EDTA-1.0 compared with V_2_O_5_. However, the bond distances of V–O–V are significantly reduced. This indicates that the incorporation of EDTA has an impact on the local atomic configuration of the material, resulting in alterations to the structural characteristics of V–O–V. This is further substantiated by the observation that the *k*^3^*–χ*(*k*) oscillation curves of the samples within the range of 6–14 Å^−1^ exhibit a distinct shape compared with those of V_2_O_5_ (Fig. 3g and Fig. S32) [36].

**Figure 4.**
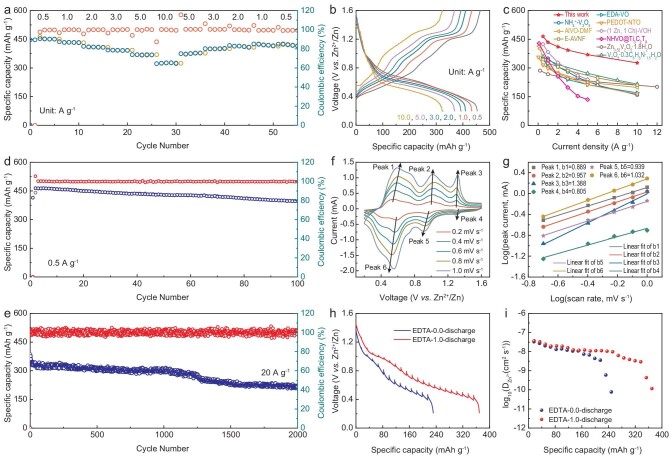
(a) Rate performance at current densities from 0.5 to 10.0 A g^−1^. (b) Galvanostatic charge-discharge profiles of EDTA-1.0 cathode. (c) Comparison between our EDTA-1.0 cathode and previous reports [29,30,35,37–42]. Cycling performance and the corresponding coulombic efficiency at (d) 0.5 and (e) 20 A g^−1^. (f) CV curves at scan rates of 0.2–1.0 mV s^−1^. (g) Log(*i*) versus log(*v*) plots at specific peak currents. (h) Discharge GITT curves and (i) the corresponding Zn^2+^ diffusion coefficients (D_Zn_^2+^).

The electrochemical performance of button batteries that are assembled by using V_2_O_5_ cathode materials intercalated with EDTA was characterized. There is good wettability between the electrodes and the electrolyte, and the electrode shows excellent stability in the electrolyte (Figs S33 and S34). Figure 4a shows the specific capacities of the EDTA-1.0 cathode at 0.5, 1.0, 2.0, 3.0, 5.0 and 10 A g^−1^, recorded as 464.6, 434, 410.9, 393, 369.3 and 324.4 mAh g^−1^, respectively. The EDTA-1.0 cathode exhibits a capacity retainment of 70.3% at 10 A g^−1^. Subsequently, when the current density is reduced to 0.5 A g^−1^, the cathode maintains a capacity of 427.3 mAh g^−1^. This indicates that the EDTA-1.0 electrode exhibits excellent rate performance. Figure 4b shows the corresponding voltage profiles. Meanwhile, the specific capacity values of different EDTA contents varied, with EDTA-1.0 exhibiting the optimal capacity (Fig. S35). Compared with recent studies on AZIBs, the EDTA-1.0 electrode demonstrates superior performance (Fig. 4c). The specific capacity of the EDTA-1.0 electrode is as high as 464.6 mAh g^−1^ at 0.5 A g^−1^ (Fig. 4d). Over 100 cycles, the capacity of 397.7 mAh g^−1^ is retained, with a coulombic efficiency of 99.8%. When the current density is boosted by a factor of 40 (20 A g^−1^), the battery still has a specific capacity of 215.2 mAh g^−1^. Notably, the capacity retention of the battery reaches 78% after 2000 discharge/charge cycles (Fig. 4e, Fig. S36 and Table S6). This indicates that the intercalation of EDTA enhanced the stability of the material; therefore, the EDTA-1.0 electrode exhibits excellent multiplicity performance and cycling stability. Multiscan-rate CV measurements were performed to explore the electrochemical kinetics of the EDTA-1.0 cathode. As shown in Fig. 4f, two pairs of redox peaks are observed at between 0.2 and 1.2 V, corresponding to the redox reactions of V^5+^/V^4+^ and V^4+^/V^3+^, respectively [43]. This result is comparable to that observed for V_2_O_5_ (Fig. S37). The peak between 1.2 and 1.6 V is related to the coordination of Zn^2+^ with EDTA (Fig. S42) [44]. The conventional equation, *i* = *av^b^*, describes the relationship between the peak current (*i*) and the scan rate (*v*). Figure 4g shows that the value of *b* is close to 1, indicating pseudocapacitive behavior in the electrochemical kinetics of the EDTA-1.0 electrode. The diffusion coefficients of D_Zn_^2+^ in the EDTA-0.0 and EDTA-1.0 cathodes were measured by using GITT (Fig. 4h and i). The D_Zn_^2+^ values in the EDTA-1.0 cathode were measured to be in the range of 10^−7^–10^−9^ cm^2^ s^−1^, which is higher than those of the EDTA-0.0 cathode (10^−7^–10^−10^ cm^2^ s^−1^). Simultaneously, it also demonstrates superiority among the reported AZIBs cathodes (Table S7). This suggests that EDTA-1.0 exhibits more rapid electrochemical kinetics. This is because the negatively charged ligand attenuated the electrostatic interactions of Zn^2+^ and the intercalation of EDTA increased the interlayer gap of the lamellar structure.

**Figure 5.**
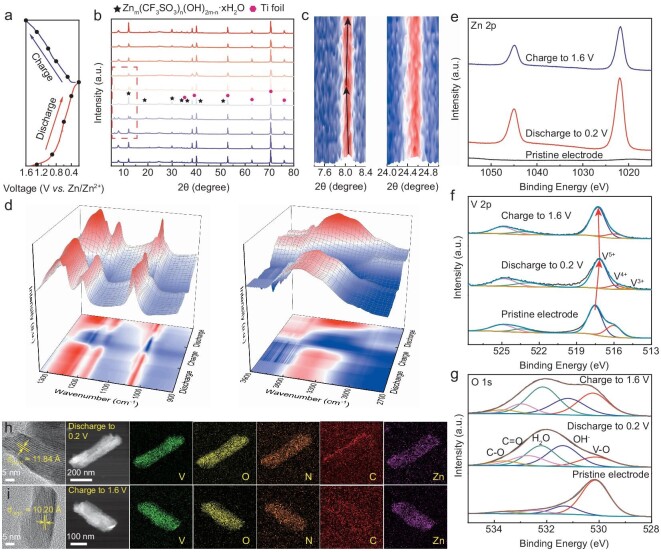
(a) Charge/discharge curves of EDTA-1.0 cathode, (b) corresponding *ex situ* XRD results, (c) *in situ* XRD results and (d) *in situ* FTIR of EDTA-1.0 cathode. High-resolution *ex situ* XPS spectra of (e) Zn 2p, (f) V 2p, (g) O 1s at the pristine, discharge to 0.2 V and the following charging to 1.6 V states. (h) and (i) HRTEM and corresponding EDS elemental mapping images of EDTA-1.0 including V, O, N, C and Zn elements at discharge to 0.2 V and the following charging to 1.6 V states.

The energy-storage mechanism of the EDTA-1.0 electrode was further investigated by using a series of *in situ* and *ex situ* tests. During the first cycle of discharge/charge, *ex situ* XRD analysis revealed the emergence of peaks at specific voltages and the presence of Zn*_m_*(CF_3_SO_3_)*_n_*(OH)_2_*_m_*_–_*_n_*·*x*H_2_O (Fig. 5a and b) [33]. *In situ* XRD analysis reveals that the (001) crystal plane shifted during discharge because of the intercalation of Zn^2+^, which increased the lattice spacing. The lattice spacing is gradually restored as Zn^2+^ is dislodged during charge, indicating the excellent reversibility of the electrode material (Fig. 5c and Fig. S38). Additionally, the intercalation and deintercalation of Zn^2+^ affect the stretching vibrations of V–O (Fig. S39). The interfacial response of the electrode material was further investigated by using attenuated total reflection surface enhanced infrared spectroscopy. As shown in Fig. S40, the spectrum at the electrode material interface changes reversibly during discharge/charge. The observed fluctuations in the O–H stretching pattern at ∼3650–3000 cm^−1^ can be attributed to proton intercalation in the electrode material and the release of –OH groups due to the decomposition of H_2_O during discharge [45]. At the same time, the increased peak intensities at ∼1246 (CF_3_), 1178 (CF_3_) and 1028 (SO_3_^2−^) cm^–1^ are due to the formation of by-products on the electrode surface during discharge (Fig. 5d) [33]. The intercalation and deintercalation of Zn^2+^ during discharge/charge also affect the valence of the electrode material. The change in the intensity of the Zn 2p peak is related to the intercalation and deintercalation of Zn^2+^ in the electrode material. The peak intensity of Zn 2p in the fully charged state is due to the partial deintercalation of Zn^2+^ (Fig. 5e and Fig. S41). During discharge, the valence of V decreases because of the intercalation of Zn^2+^. Consequently, the pentavalent vanadium peak shifted to lower energies and the trivalent vanadium peak appeared simultaneously (Fig. 5f). The changes in H_2_O and OH^−^ in the O 1s spectra are due to the generation of Zn*_m_*(CF_3_SO_3_)*_n_*(OH)_2_*_m_*_–_*_n_*·*x*H_2_O during discharge/charge (Fig. 5g). The peak at 399.3 eV is attributed to the formation of a Zn–N bond, indicating the chelation of EDTA molecules with Zn^2+^ (Fig. S42) [44]. The HRTEM findings demonstrate the reversibility of the charging and discharging process of Zn^2+^ (Fig. 5h and i). The lattice spacing on the (001) crystal plane increases to 11.84 Å under complete discharge and returns to 10.20 Å upon charging to 1.6 V. The energy-dispersive spectroscopy (EDS) elemental mapping images show a homogeneous dispersion of V, O, N, C and Zn before and after charging. Additionally, the EDX images demonstrate a reduction in the Zn content after charging (Fig. S43). The SEM images reveal that the electrode surface was covered with numerous nanosheets after complete discharge (Fig. S44). Furthermore, the number of nanosheets was significantly reduced at the end of the charging process, indicating excellent reversibility (Fig. S45).

**Figure 6.**
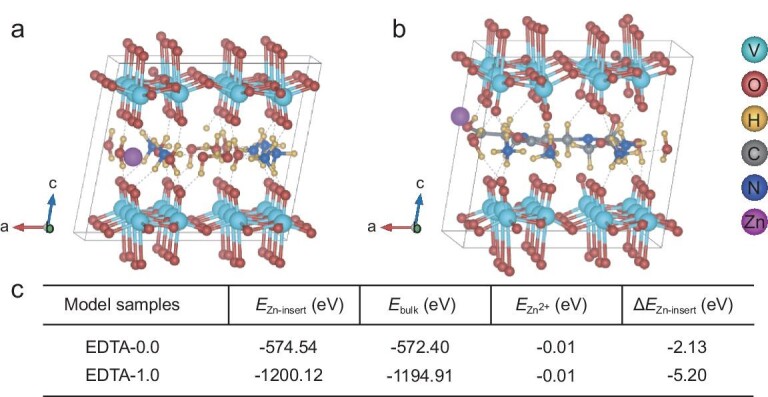
Schematic diagram of the optimized structure after inserting Zn in (a) EDTA-0.0 and (b) EDTA-1.0. (c) Calculated *E*_Zn-insert_ (eV), *E*_bulk_ (eV), *E*_Zn^2+^_ (eV) and Δ*E*_Zn-insert_ (eV) per cell for the model sample.

To further study the effect of EDTA on the structure of VO and the insertion energy of Zn^2+^, we employed density functional theory calculations to investigate the atomic structure and Zn^2+^ insertion energy of the materials. The structural model indicates that the insertion of EDTA expands the interlayer spacing of the structure, creating space for the migration of zinc ions (Fig. S46). Concurrently, the EDTA molecules that are situated between the structural layers serve as interlayer supports, stabilizing the ion shuttle channel and facilitating the rapid migration of ions (Fig. 6a and b). An analysis of the energy between the zinc ion and the electrode material structure reveals that the zinc insertion energy of EDTA-1.0 is –5.20 eV—a value that is lower than that of EDTA-0.0 (–2.13 eV) (Fig. 6c). This indicates that zinc ions are more easily stored in EDTA-1.0 due to the insertion of EDTA. Therefore, the EDTA layer, with its charged ions, increases the interlayer spacing and creates a stable interlayer structure that provides the zinc ions with stable shuttle channels and high migration rates in the layered structure. This achieves structural stability and low kinetic energy barriers for the electrode material during discharge/charge.

## CONCLUSIONS

In summary, we synthesized various nanomaterials with charged organic carboxylic acids intercalated in VO as cathodes for AZIBs. The effects of different organic carboxylic acid ligands on the structural stability, nanolayer spacing and V-element valence changes of the VO were systematically investigated and the mechanism of the role of organic carboxylic acid ligands in the zinc-ion storage process was revealed. Organic carboxylic acid intercalation of layered VO stabilized the nanolayer structure, increased the Zn^2+^ migration rate and improved the molecular dynamics. The work presented here provides new insights into improving energy storage in AZIBs by designing nanolayer structures.

## METHODS

The detailed experimental reagents and methods can be found in the Supplementary information.

## Supplementary Material

nwae336_Supplemental_File
