# Supplementary material for: Charged organic ligands inserting/supporting the nanolayer spacing of vanadium oxides for high-stability/efficiency zinc-ion batteries
Source: Natl Sci Rev. 2024 Sep 20;11(10):nwae336. doi: 10.1093/nsr/nwae336 (PMC11487576; doi:10.1093/nsr/nwae336)
Supplement: nwae336_Supplemental_File [file nwae336_supplemental_file.pdf]

Supplementary information

Charged organic ligands inserting/supporting the nanolayer spacing of vanadium oxides for high-stability/efficiency zinc ion batteries

Guoqiang Yuan[1], Yichun Su[1], Xiangling Zhang[1], Biao Gao[1], Jinliang Hu[2], Yangyang Sun[1], Wenting Li[1], Zhan Zhang[1], Mohsen Shakouri[3] & Huan Pang[1,*]

[1]School of Chemistry and Chemical Engineering, Yangzhou University, Yangzhou, 225009, P. R. China
[2]Jiangsu Yangnong Chemical Group Co. Ltd., Yangzhou, 225009, P. R. China
[3]Canadian Light Source Inc., University of Saskatchewan, Saskatoon, S7N 2V3, Canada
E-mail: huanpangchem@hotmail.com;

Section S1 Experimental Procedures

Materials and reagents

All reagents were of analytical grade and could be used without further purification. Ammonium metavanadate (NH_4VO_3, AR, 99%), Glycine (Gly, ACS, ≥ 98.5 %), DL-Alanine (DL-Ala, AR, 99 %), Oxalic acid (OA, AR, 98 %), Malonic acid (MA, AR, 99.5 %), Glutaric acid (GA, AR, 99 %), 2,5-Thiophenedicarboxylic acid (OB, AR, 98 %), Iminodiacetic acid (IDA, AR, 98 %), 1,2,3-Propanetricarboxylic Acid (TA, AR, >98 %), Citric acid (CA, AR, 98 %), Nitrilotriacetic acid (NTA, ACS, ≥ 99.0 %), Ethylenediaminetetraacetic acid (EDTA, AR, 99.5%) were supplied by Shanghai Aladdin Chemical Reagent Co., Ltd. Hydrochloric acid (HCL, AR, 37%) was obtained from Sinopharm Group Co. Ltd. All reagents used in this experiment have not undergone any additional processing or purification. Deionized water (DIW, 18.2 MΩ/cm) was used for synthetic experiments.

Synthesis of intercalated nanomaterials with Organic carboxylic Acid

In a typical synthesis, 1 mmol of ammonium metavanadate was added to 35 mL of deionized water and stirred vigorously for 1 h. The solution changed color to orange-yellow. Subsequently, 0.25 mL of HCL solution was added, causing the color to change to orange. Finally, 1 mM of organic carboxylic acids, including Gly, DL-Ala, OA, MA, GA, OB, IDA, TA, CA, NTA, and EDTA, were added. The process was thoroughly stirred to prevent precipitation. Next, the solution was transferred to a 50 mL sealed autoclave lined with polytetrafluoroethylene and kept in an oven at 160°C for 12 h. After cooling to room temperature, the samples were washed three times each with DIW and ethanol solution. Finally, the samples were obtained through freeze-drying. The samples were named None, Gly, DL-Ala, OA, MA, GA, OB, IDA, TA, CA, NTA, and EDTA based on the type of organic carboxylic acid.

Synthesis of intercalated nanomaterials with varying amounts of organic carboxylic acids

In a typical synthesis, 1 mmol of ammonium metavanadate was added to 35 mL of deionized water and stirred vigorously for 1 h. The solution changed color to orange-yellow. Subsequently, 0.25 mL of HCL solution was added, causing the color to change to orange. Add 0, 1, 2.5, and 5 mM EDTA, stirring thoroughly to prevent precipitation. Next, the solution was transferred to a 50 mL sealed autoclave lined with polytetrafluoroethylene and kept in an oven at 160 °C for 12 h. After cooling to room temperature, the samples were washed three times each with DIW and ethanol solution. Finally, the samples were obtained through freeze-drying. The samples were named as EDTA-0.0, EDTA-1.0, EDTA-2.5, and EDTA-5.0 based on their EDTA content.

Material Characterization

The morphology of samples was observed with Zeiss_Supra 55 microscope scanning electron microscope (SEM, 5 kV) and Hitachi HT7800 transmission electron microscope (TEM, 120 kV). High-Resolution Transmission Electron Microscope (HRTEM), selected area electron diffraction (SAED) images, and Energy Dispersive X-ray (EDX) mapping were performed on a Tecnai G2 F30 S-TWIN transmission electron microscopy with an accelerating voltage of 300 kV and a FEI-Talos F200X transmission electron microscopy with an accelerating voltage of 200 kV. X-ray diffraction (XRD) and in-situ XRD data were obtained using a MiniFlex-600X (Rigaku) with Cu Kα radiation (λ = 1.5406 Å). Fourier Transform Infrared (FTIR) measurements use Cary 610/670 (Varian Co.). Raman and in-situ Raman spectra were obtained using a Renishaw in Via Raman microscope with an excitation length of 532 nm. Thermogravimetry analysis (TGA) was performed under an N_2 atmosphere from room temperature to 600 □ at a heating rate of 5 □ min^{-1} by a TGA Q50 (TA instrument). The C, N, and H elements determination passed the Vario EL cube analyzer from Elementar. X-ray photoelectron spectra (XPS) were collected using a Thermo Scientific

ESCALAB 250Xi X-ray photoelectron spectrometer. Autosorb-Iq obtained the N_2 adsorption-desorption isotherms via Brunauer-Emmet-Teller (BET) method. Water contact angle was performed on Kruss Tensiometer (K100MK2, Germany). The UV-vis diffuse reflectance spectra (UV-vis DRS) were obtained for the dry-pressed disk samples using a Cary 500 Scan UV-vis spectrophotometer (Varian). The X-ray absorption find structure (XAFS) spectra (V K-edge) were collected at Saskatoon, Canada with Canadian Light Source using SXRMB beam line. The XAFS data were processed according to the standard procedures using the Athena module implemented in the IFEFFIT software packages. The EXAFS spectra were obtained by subtracting the post-edge background from the overall absorption and then normalizing with respect to the edge-jump step. Subsequently, the $\chi(k)$ data of were Fourier transformed to real (R) space using a hanning windows (dk = 1.0 $Å^{-1}$) to separate the EXAFS contributions from different coordination shells. To obtain the quantitative structural parameters around central atoms, least-squares curve parameter fitting was performed using the ARTEMIS module of IFEFFIT software packages [1-2]. For Wavelet Transform analysis, the χ(k) exported from Athena was imported into the Hama Fortran code [3]. The parameters were listed as follow: R-range, 0.0 - 4.0 Å, k-range, 0 - 15.0 $Å^{-1}$ for sample and Standards; k weight, 2; and Morlet function with $\kappa = 9$, $\sigma = 1$ was used as the mother wavelet to provide the overall distribution.

Electrochemical Characterization

The electrochemical performance tests were conducted using CR-2032 coin cell batteries. The super P carbon and polyvinylidene difluoride were mixed with the active material in an NMP solution at a mass ratio of 7:2:1. The resulting mixture was then coated onto the titanium foil and the coated foil was dried overnight at 60°C in a vacuum drying oven to prepare the working electrode. The active substance was loaded within the range of 0.5-0.9 mg. The button cell was assembled using the above electrode as the

positive electrode, zinc metal foil as the negative electrode, 3 M $(CF_3SO_3)_2Zn$ aqueous solution as the electrolyte, and glass fiber (GF/D grade) as the diaphragm. Constant current discharge/charge, multiplier performance, and constant current intermittent titration technique (GITT) tests were conducted at room temperature using the Blue Battery Test System (Wuhan, China). The test voltage window ranged from 0.2 to 1.6 V. The GITT test was conducted using a current density of 100 mA g^{-1}. The diffusion coefficient was calculated using the following formula:

$$D = \frac{4}{\pi\tau}\left(\frac{n_m v_m}{S}\right)^2 \left(\frac{\Delta E_S}{\Delta E_\tau}\right)^2$$

Where, τ is the current pulse duration (1 min); n_m is the quantity of moles; v_m is the molar volume; S is the interface area between electrode and electrolyte (served as the geometric area of the electrode); ΔE_s and ΔE_τ are the steady-state voltage change and the overall voltage change after adding a current pulse in a cycle of GITT test[4]. Cyclic voltammetry (CV) and electrochemical impedance spectroscopy (EIS) tests were conducted using an electrochemical workstation (CHI 760E, China).

DFT calculations

The molecular geometries were optimized by Gaussian 09 with a functional of B3LYP and a basis of 6-31G(d,p)[5]. The calculated results of ESP were carried out by a wavefunction analysis tool Multiwfn[6]. The spin-polarized density functional theories (DFT) were carried out by using the Vienna Ab initio Simulation Package (VASP)[7-8]. The Perdew−Burke−Ernerhof exchange correlation functional was used in conjunction with periodic boundary spin-polarized DFT + U method[9]. The DFT + U implemented in VASP following Dudarev's approach was employed to add on-site potentials to the d electrons of V with a value of 3.2 eV[10-11]. The D3 correction of Grimme was adopted to compensate for the lack of van der Waals interaction description in the GGA functional[12]. The energy cutoff was set to 450 eV. The energy convergence was set to 10^{-5} eV and the force convergence criterion was set to 0.03 eV·$Å^{-1}$. The Monkhorst-

Pack k-points grid was set to be 3×5×3 and 3×2×3 for NH_4 and EDTA intercalated V_2O_5 during the calculations.

In-situ FTIR Experiments

The in-situ FTIR measurements are performed at room temperature using a PerkinElmer Spectrum 3 equipped with a MCT (mercury-cadmium-telluride) detector cooled by liquid nitrogen. The test spectrum has a scanning range of 4000 cm^{-1} to 600 cm^{-1}, with a resolution of 4 cm^{-1}. In-situ tests were conducted utilising the electrochemical cell mould produced by Shanghai Yuanfang Technology Co. To monitor changes during charging and discharging, a zinc selenide half-cylinder was employed as the reflector. During the test, the assembled in-situ cell mould (devoid of electrode material) was scanned as a background, and the initial spectrum (devoid of current) was collected. Following a sufficient period of rest for the cell, the spectra during the charging and discharging processes were collected.

Section S2 Results and discussion

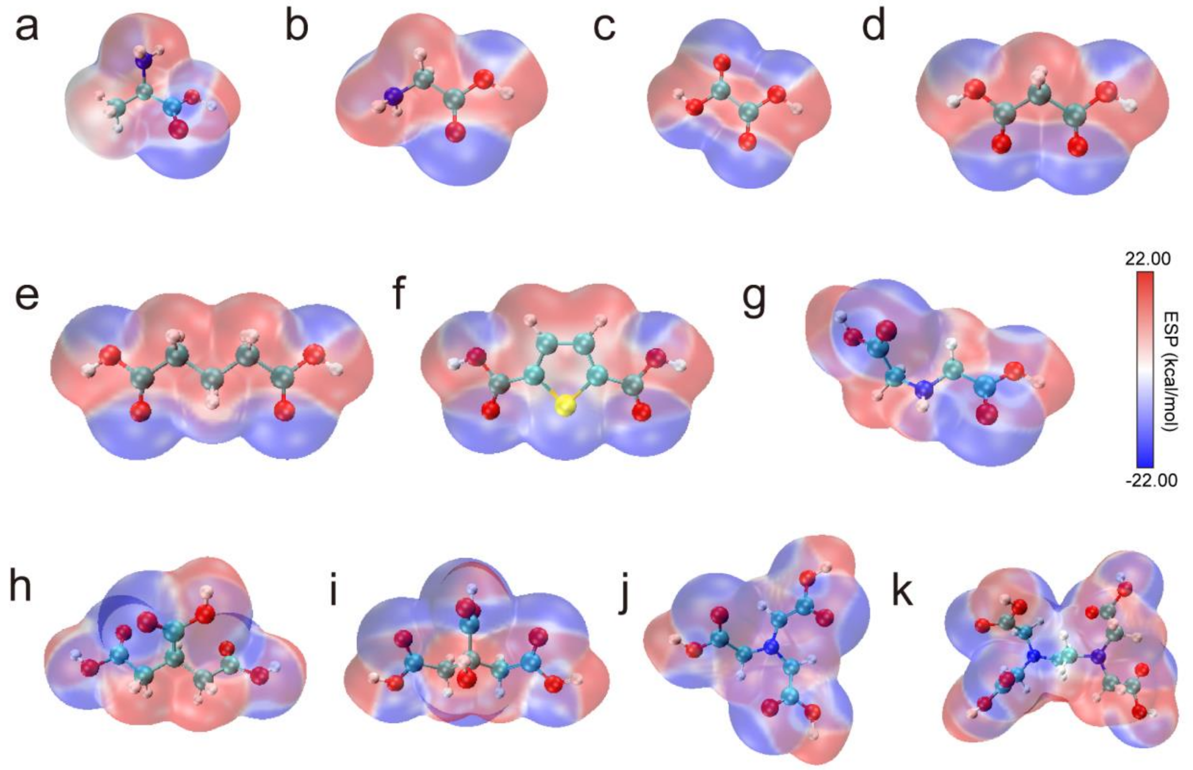

Figure. S1 Electrostatic potential distribution map of (a) Gly, (b) DL-Ala, (c) OA, (d) MA, (e) GA, (f) OB, (g) IDA, (h) TA, (i) CA, (j) NTA, (k) EDTA.

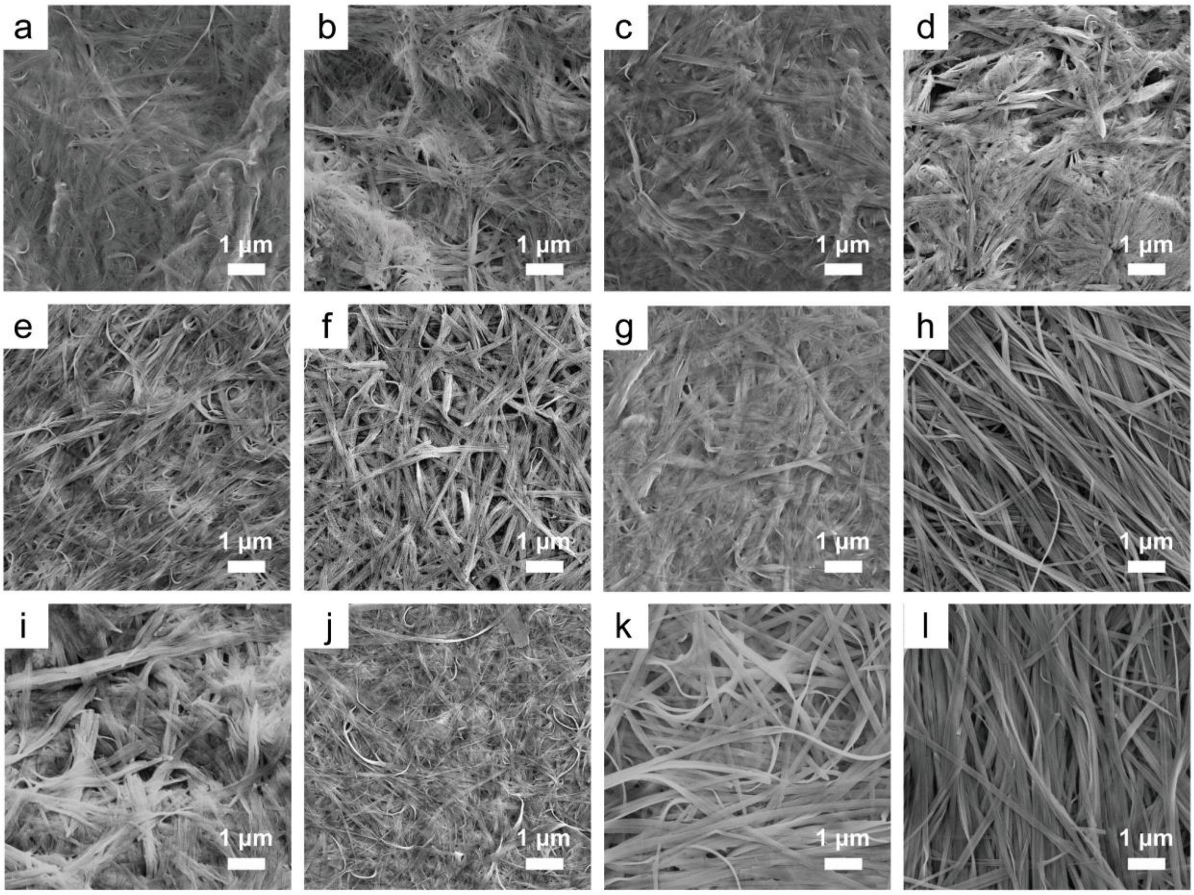

Figure. S2 SEM images of (a) None, (b) Gly, (c) DL-Ala, (d) OA, (e) MA, (f) GA, (g) OB, (h) IDA, (i) TA, (j) CA, (k) NTA, (l) EDTA.

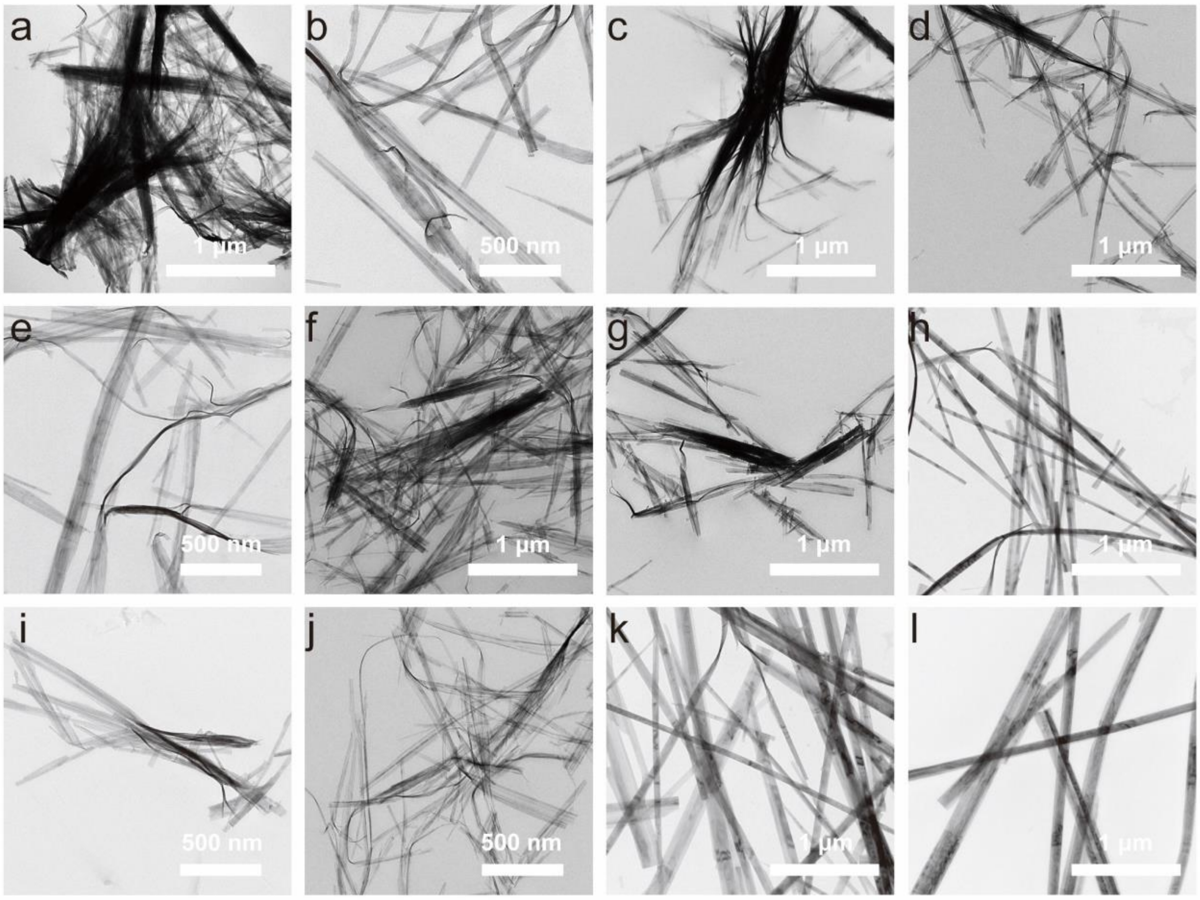

Figure. S3 TEM images of (a) None, (b) Gly, (c) DL-Ala, (d) OA, (e) MA, (f) GA, (g) OB, (h) IDA, (i) TA, (j) CA, (k) NTA, (l) EDTA.

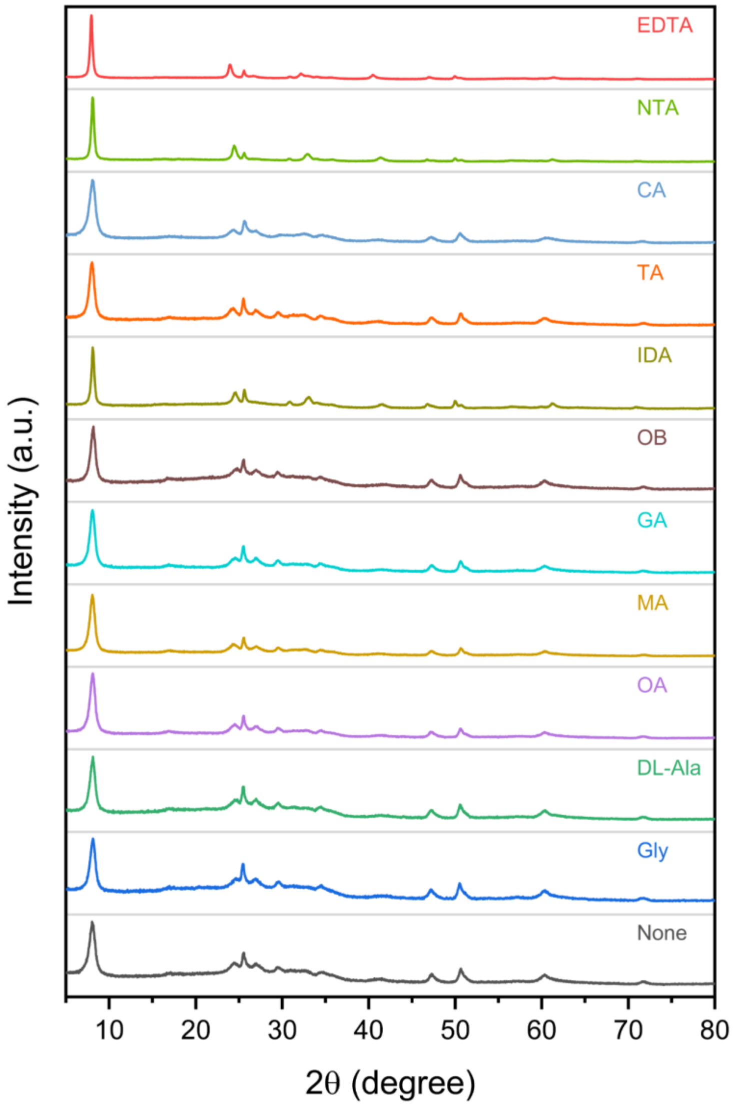

Figure. S4 XRD patterns of None, Gly, DL-Ala, OA, MA, GA, OB, IDA, TA, CA, NTA, EDTA.

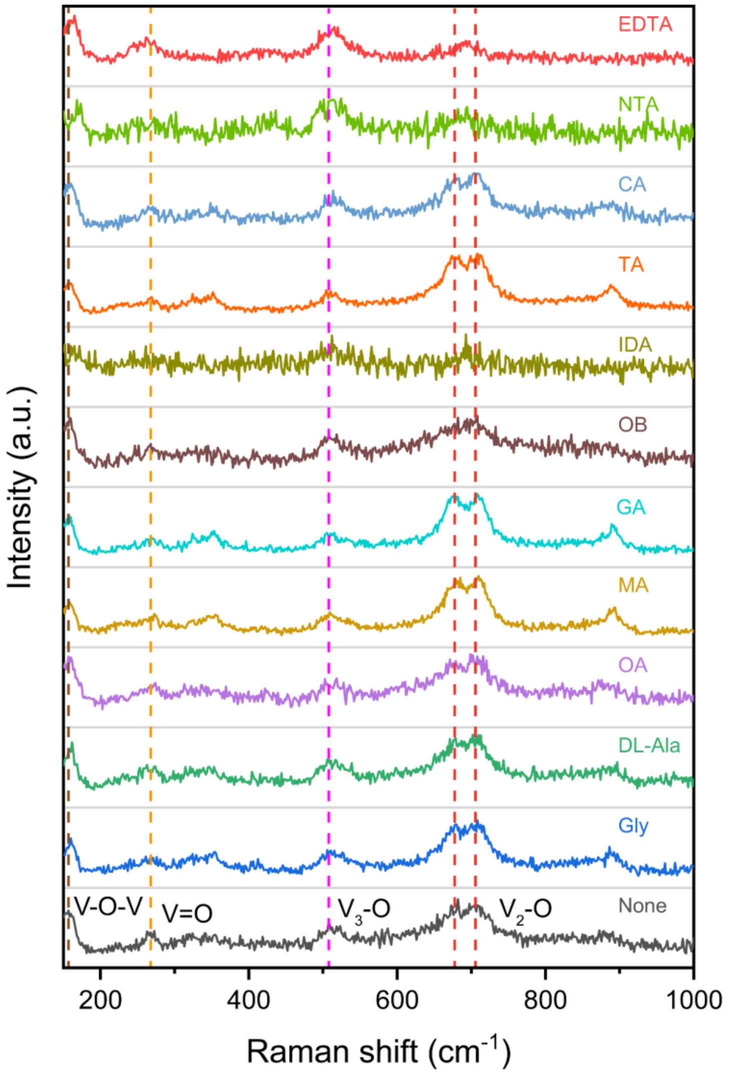

Figure. S5 The Raman spectra of None, Gly, DL-Ala, OA, MA, GA, OB, IDA, TA, CA, NTA, EDTA.

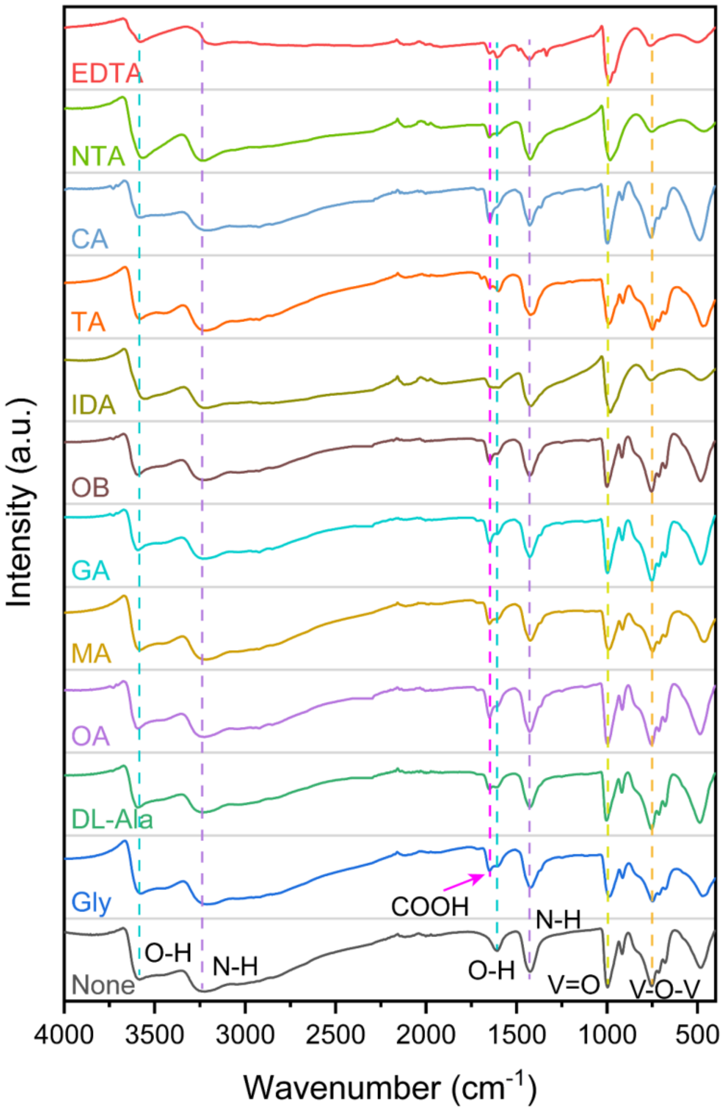

Figure. S6 FTIR spectra of None, Gly, DL-Ala, OA, MA, GA, OB, IDA, TA, CA, NTA, EDTA.

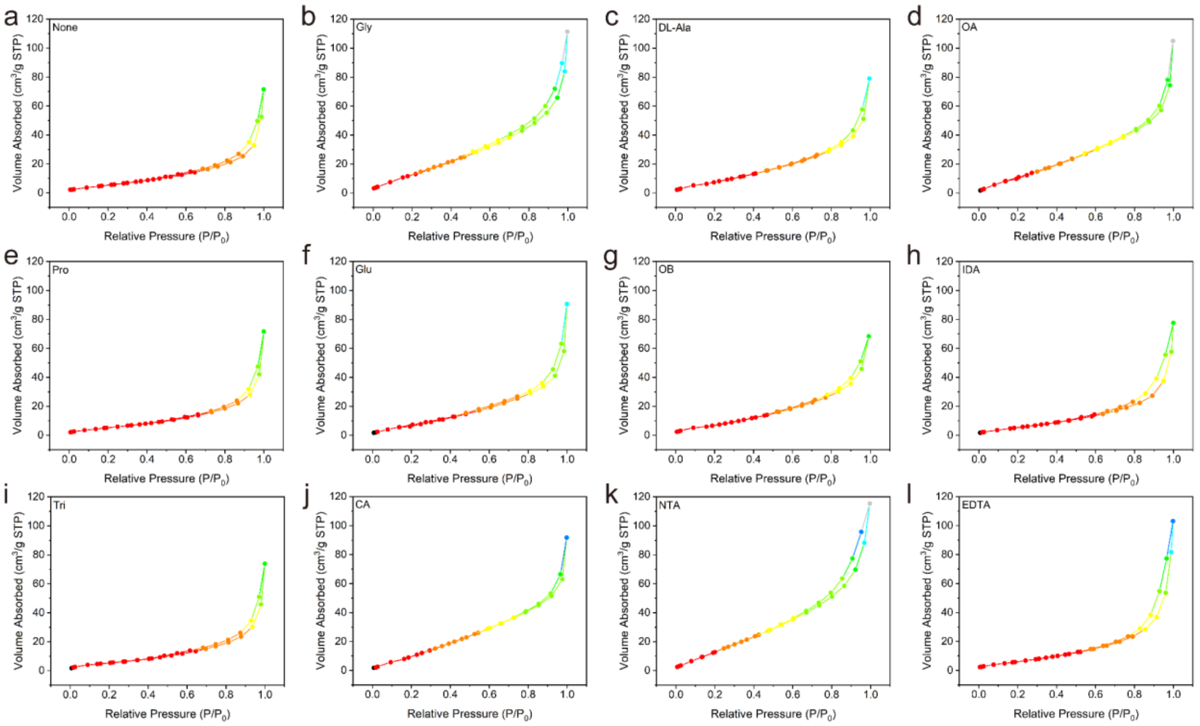

Figure. S7 Nitrogen absorption isotherm of (a) None, (b) Gly, (c) DL-Ala, (d) OA, (e) MA, (f) GA, (g) OB, (h) IDA, (i) TA, (j) CA, (k) NTA, (l) EDTA.

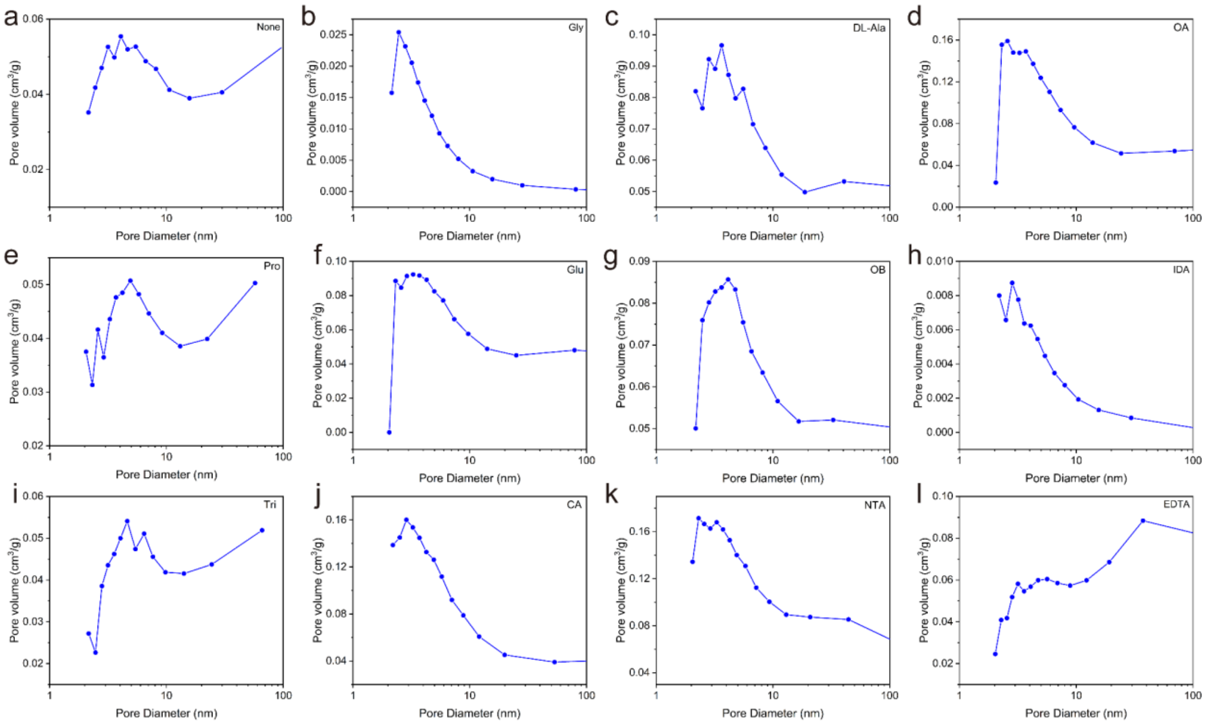

Figure. S8 Pore volume distribution of (a) None, (b) Gly, (c) DL-Ala, (d) OA, (e) MA, (f) GA, (g) OB, (h) IDA, (i) TA, (j) CA, (k) NTA, (l) EDTA.

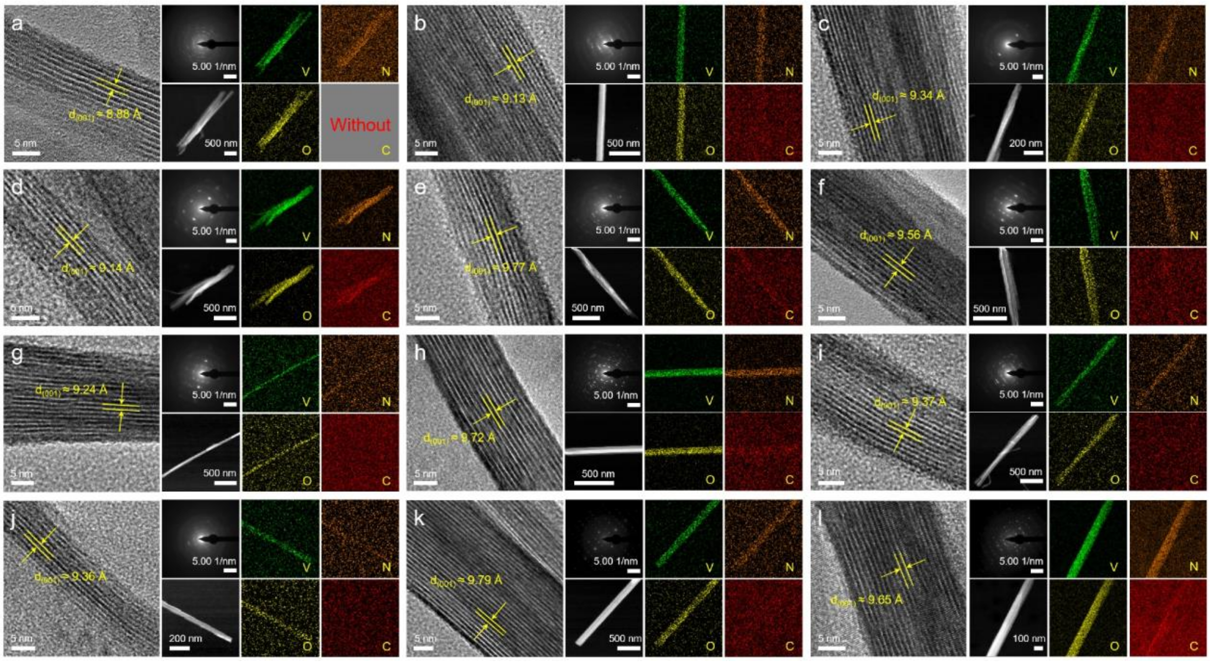

Figure. S9 HRTEM, SAED, and corresponding EDS elemental mapping images of (a) None, (b) Gly, (c) DL-Ala, (d) OA, (e) MA, (f) GA, (g) OB, (h) IDA, (i) TA, (j) CA, (k) NTA, (l) EDTA.

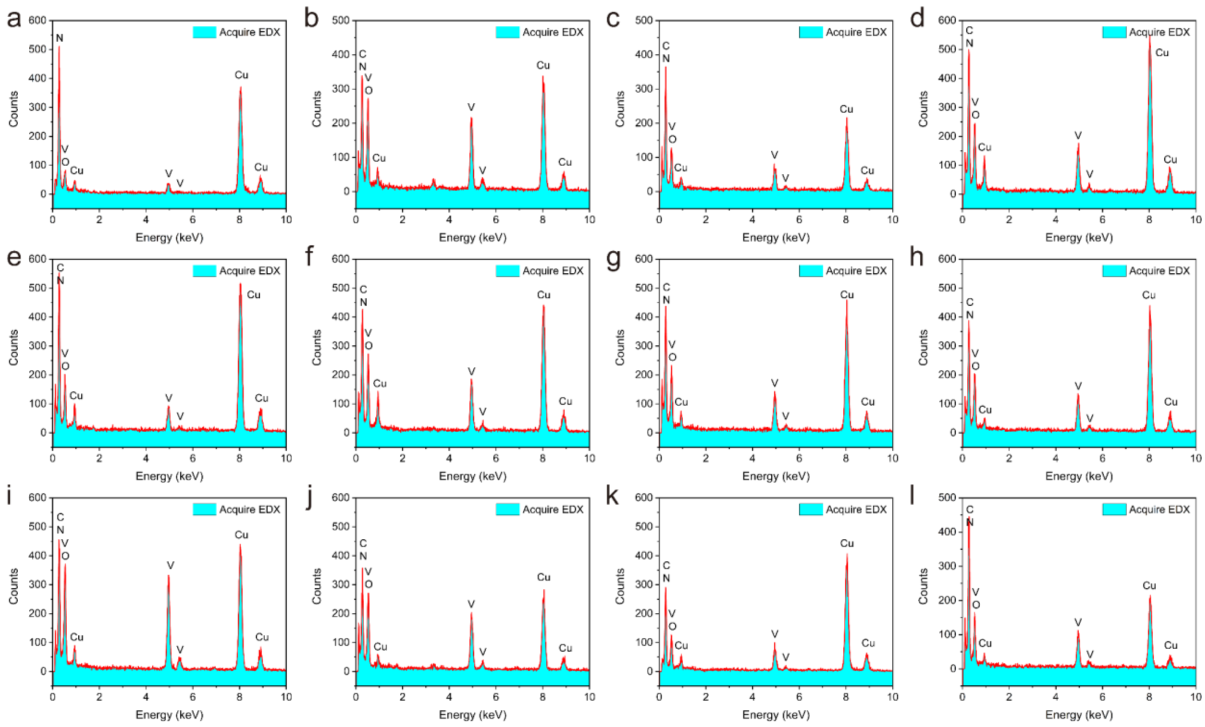

Figure. S10 EDX images of (a) None, (b) Gly, (c) DL-Ala, (d) OA, (e) MA, (f) GA, (g) OB, (h) IDA, (i) TA, (j) CA, (k) NTA, (l) EDTA.

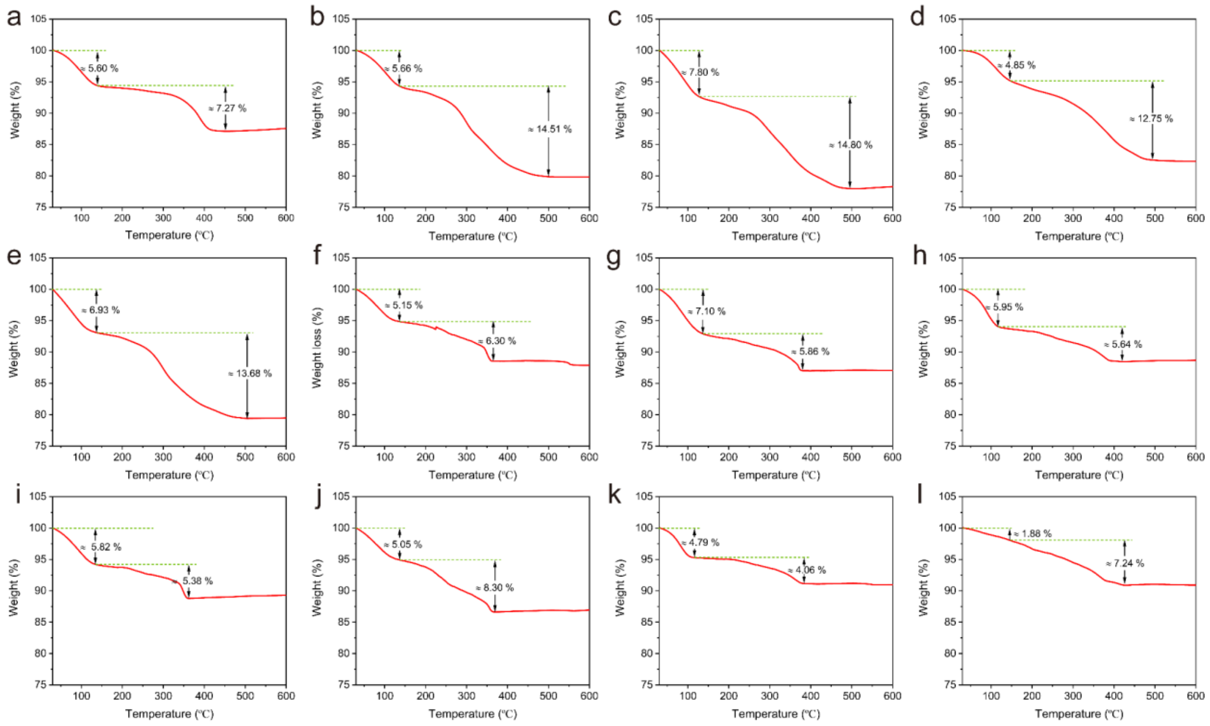

Figure. S11 The TGA curves of (a) None, (b) Gly, (c) DL-Ala, (d) OA, (e) MA, (f) GA, (g) OB, (h) IDA, (i) TA, (j) CA, (k) NTA, (l) EDTA.

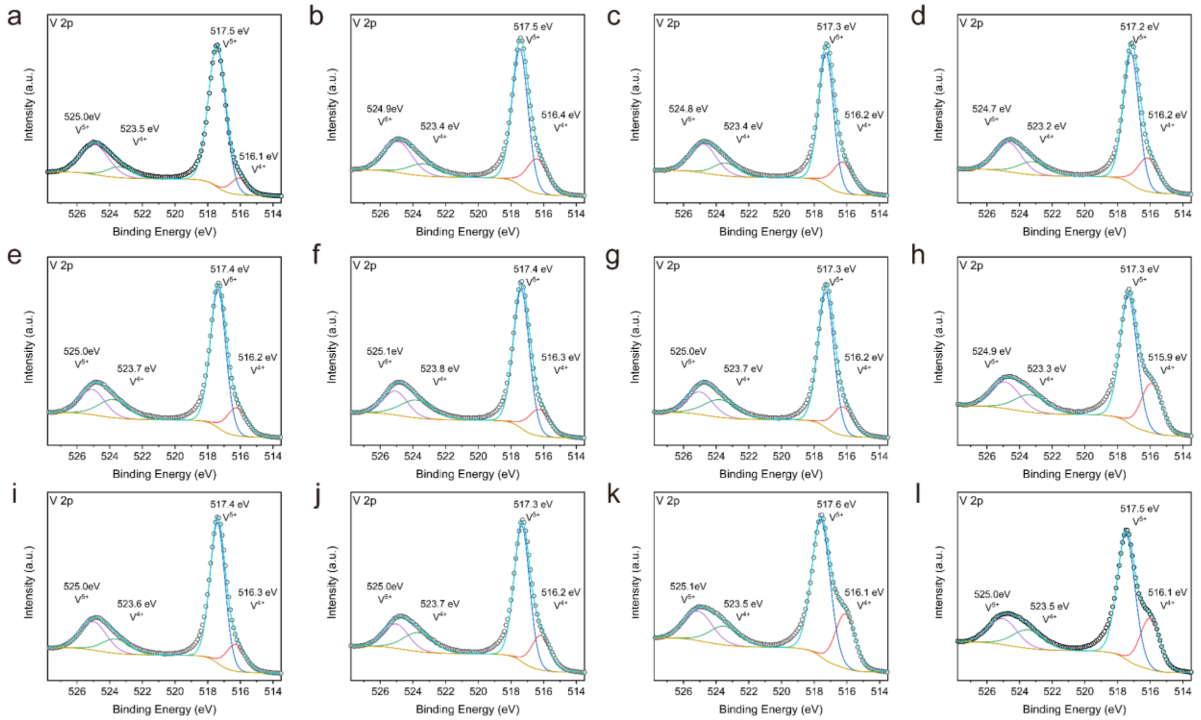

Figure. S12 The V 2p spectra of (a) None, (b) Gly, (c) DL-Ala, (d) OA, (e) MA, (f) GA, (g) OB, (h) IDA, (i) TA, (j) CA, (k) NTA, (l) EDTA.

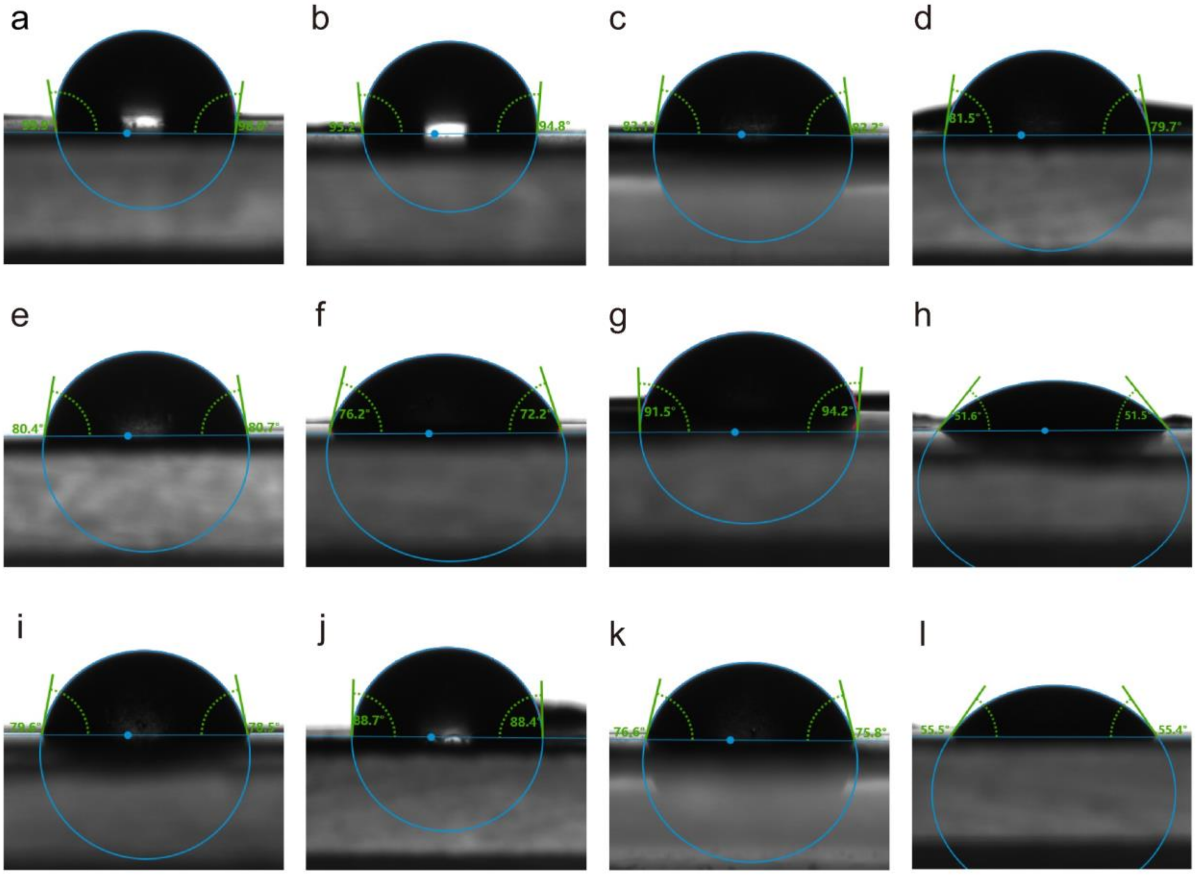

Figure. S13 The water contact angle of (a) None, (b) Gly, (c) DL-Ala, (d) OA, (e) MA, (f) GA, (g) OB, (h) IDA, (i) TA, (j) CA, (k) NTA, (l) EDTA.

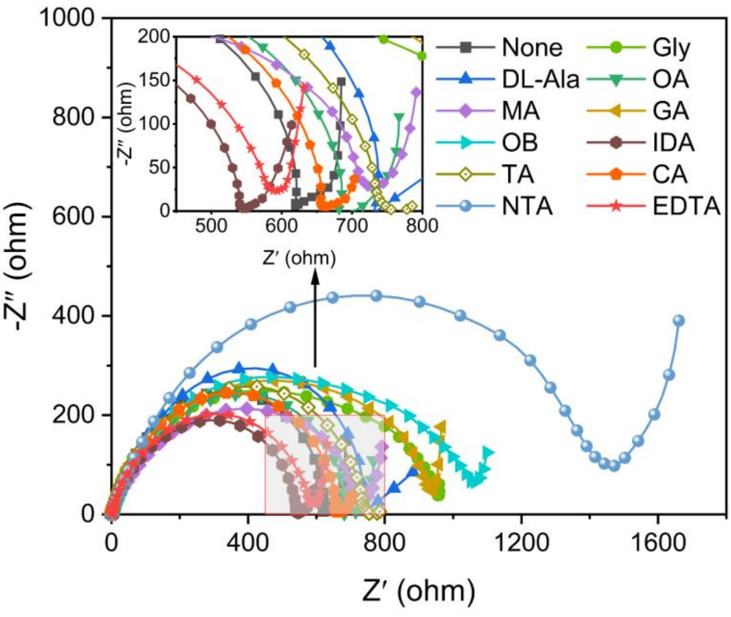

Figure. S14 EIS spectra of different electrodes and the enlarged EIS spectra (insert).

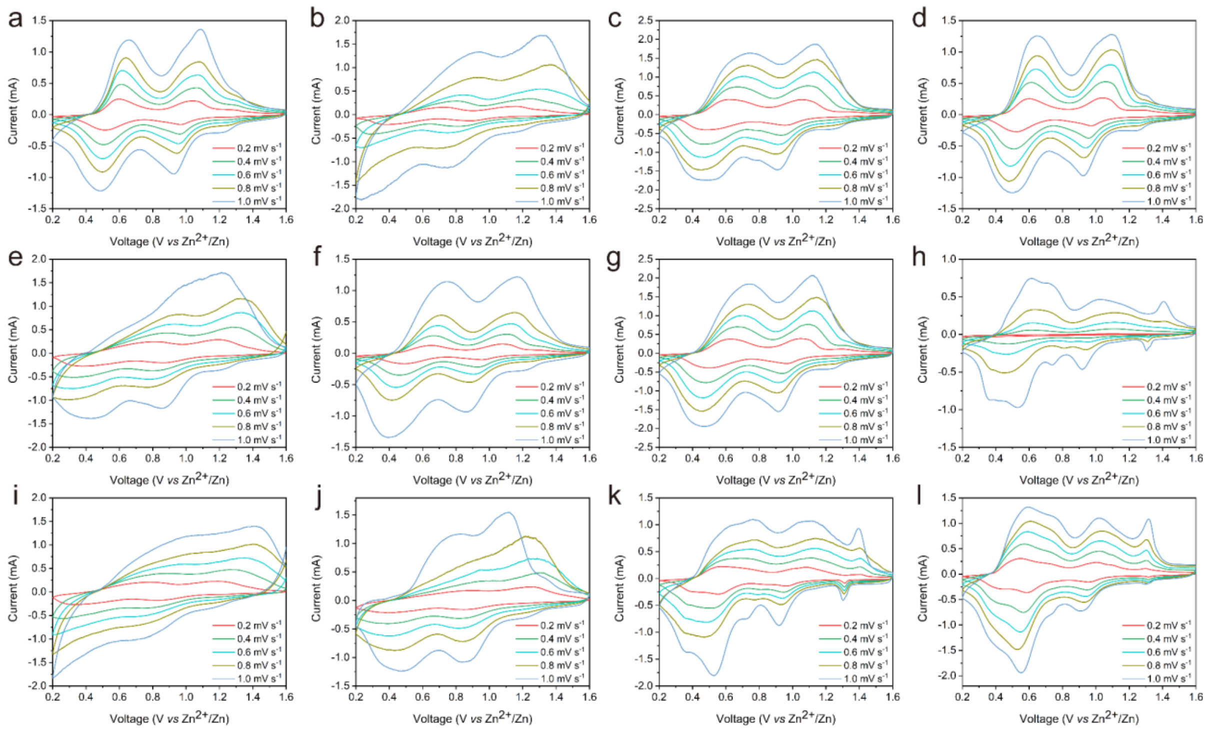

Figure. S15 The CV curves at different scan rates of (a) None, (b) Gly, (c) DL-Ala, (d) OA, (e) MA, (f) GA, (g) OB, (h) IDA, (i) TA, (j) CA, (k) NTA, (l) EDTA.

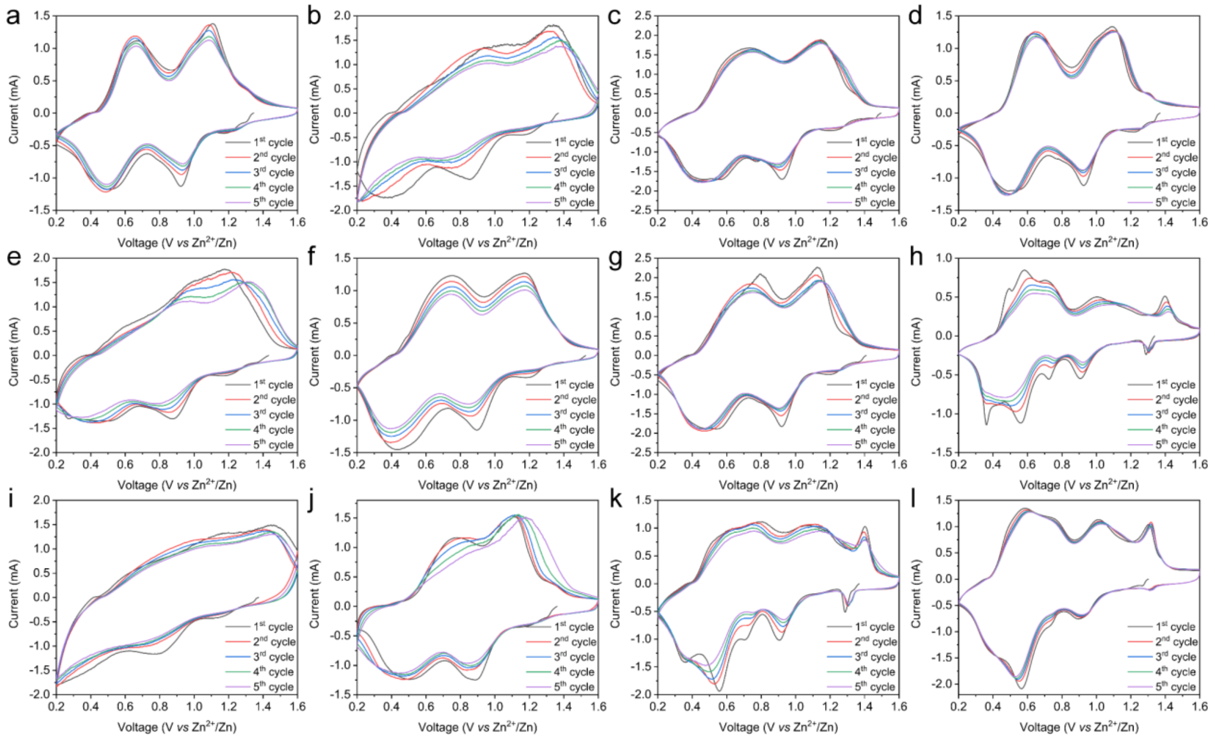

Figure. S16 The CV curves at 1.0 mV s^{-1} scan rate of (a) None, (b) Gly, (c) DL-Ala, (d) OA, (e) MA, (f) GA, (g) OB, (h) IDA, (i) TA, (j) CA, (k) NTA, (l) EDTA.

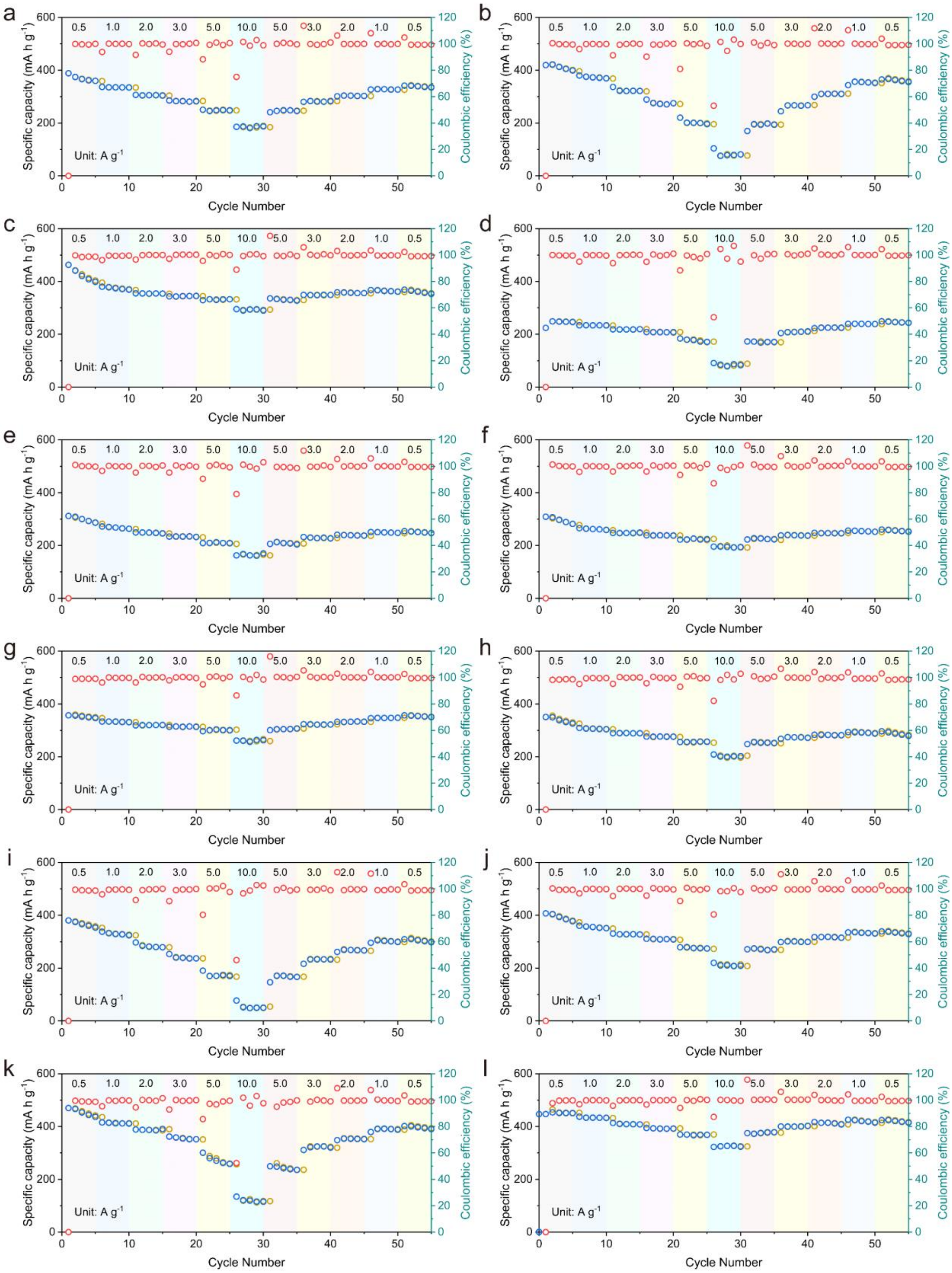

Figure. S17 Rate performances at the current density from 0.5 A g^{-1} to 10.0 A g^{-1} of (a) None, (b) Gly, (c) DL-Ala, (d) OA, (e) MA, (f) GA, (g) OB, (h) IDA, (i) TA, (j) CA, (k) NTA, (l) EDTA.

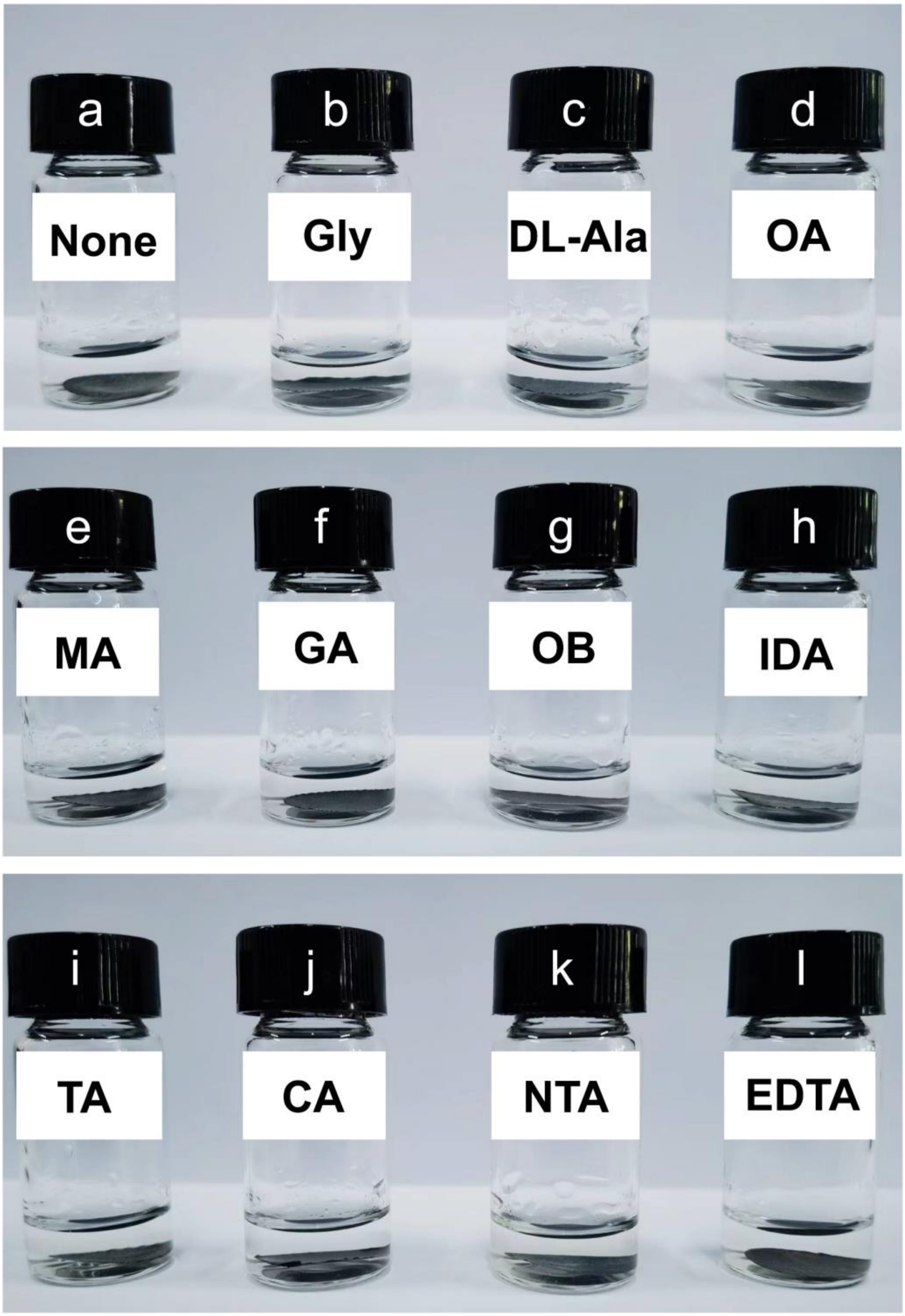

Figure. S18 The optical pictures of (a) None, (b) Gly, (c) DL-Ala, (d) OA, (e) MA, (f) GA, (g) OB, (h) IDA, (i) TA, (j) CA, (k) NTA, (l) EDTA electrode soaked in electrolyte for one month.

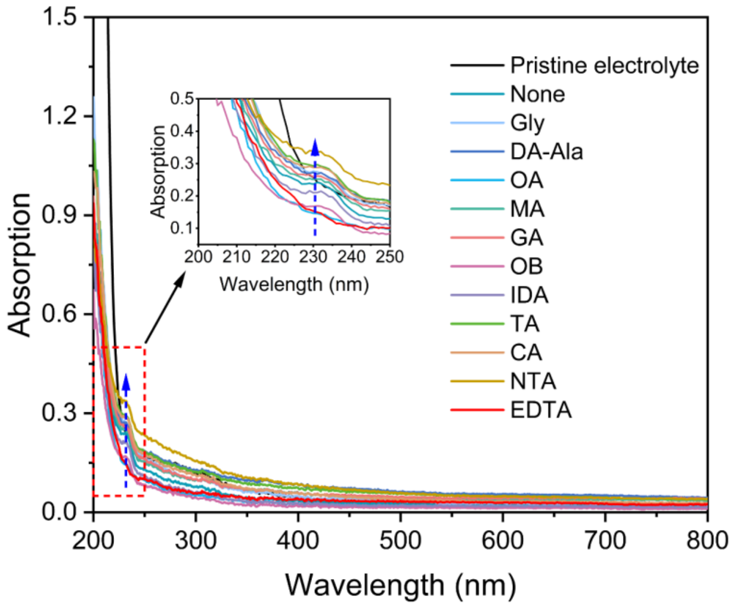

Figure. S19 UV-vis spectra of pristine electrolyte and electrolytes after electrodes soak.

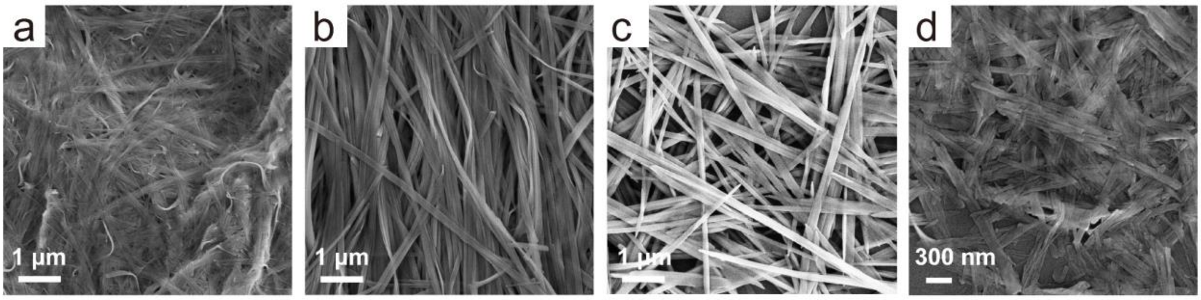

Figure. S20 SEM images of (a) EDTA-0.0, (b) EDTA-1.0, (c) EDTA-2.5, (d) EDTA-5.0.

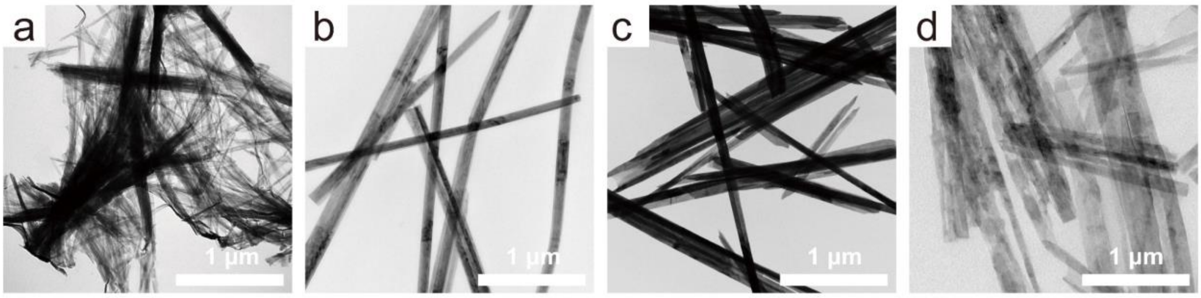

Figure. S21 TEM images of (a) EDTA-0.0, (b) EDTA-1.0, (c) EDTA-2.5, (d) EDTA-5.0.

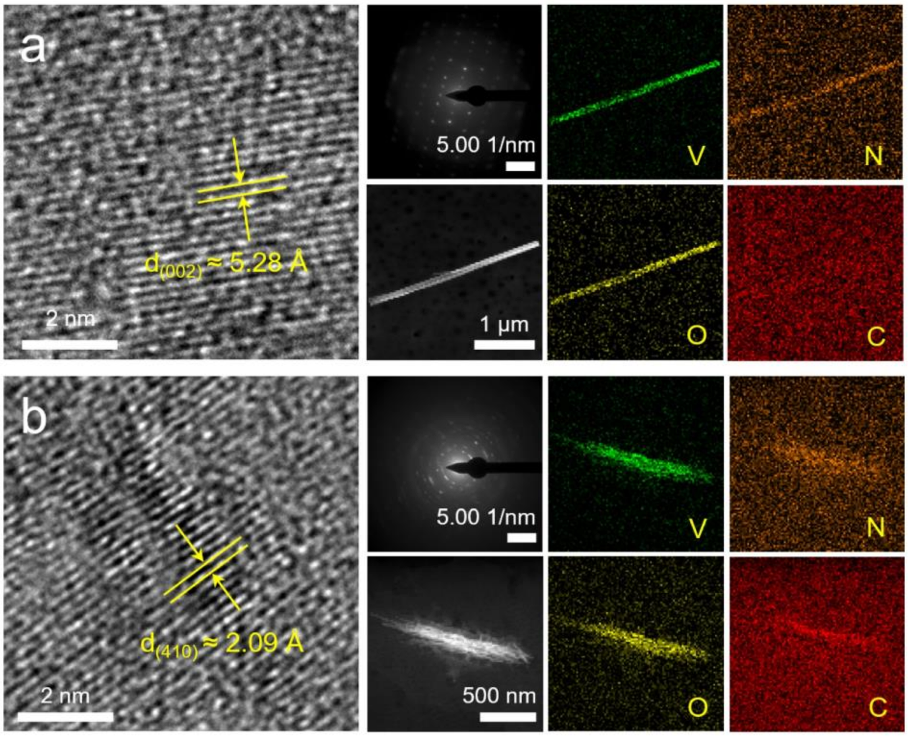

Figure. S22 HRTEM, SAED, and corresponding EDS elemental mapping images of (a) EDTA-2.5 and (b) EDTA-5.0.

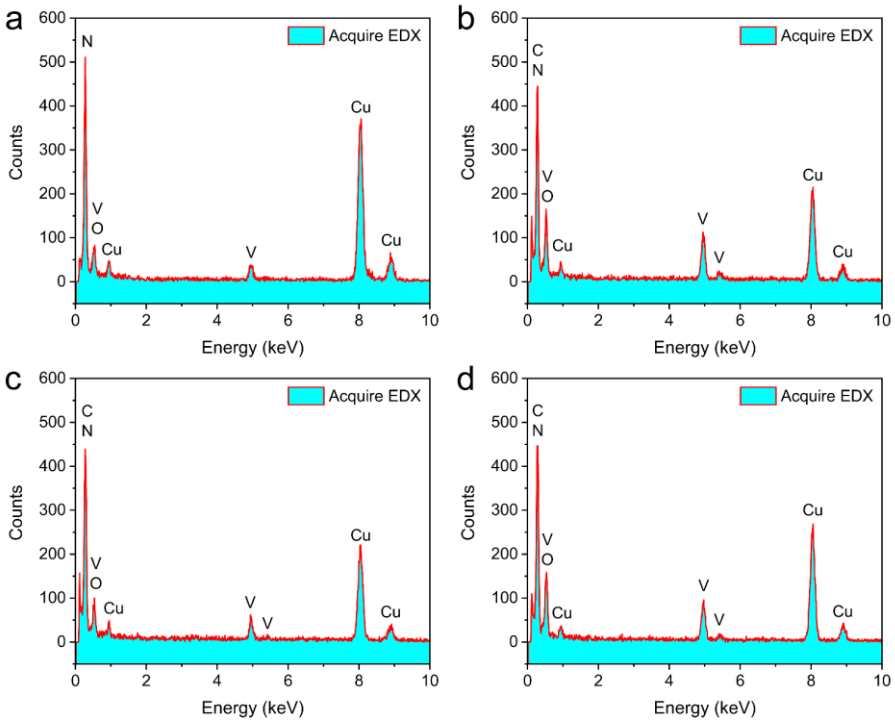

Figure. S23 EDX images of (a) EDTA-0.0, (b) EDTA-1.0, (c) EDTA-2.5 and (d) EDTA-5.0.

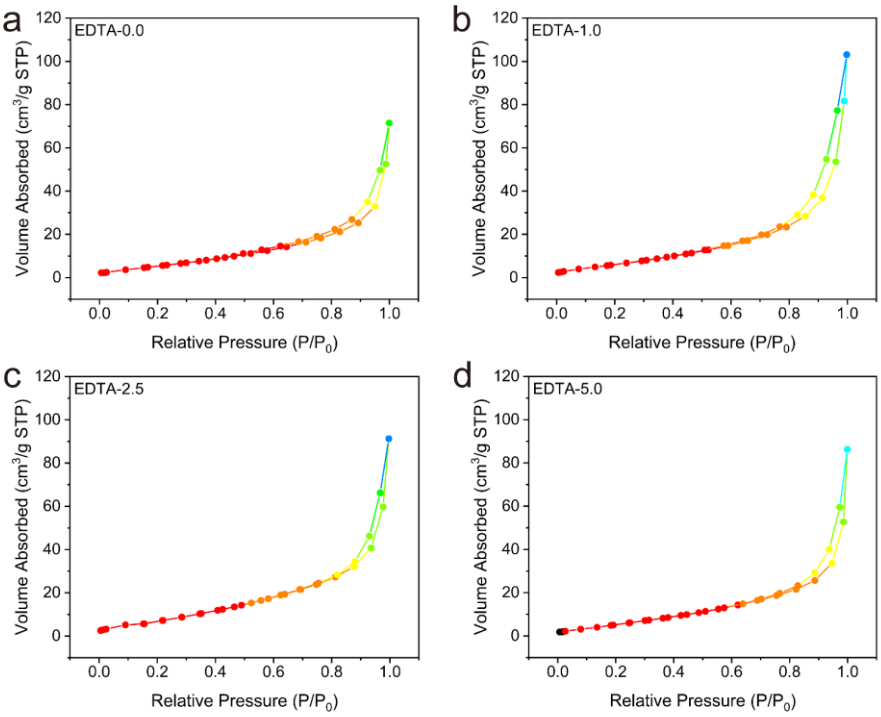

Figure. S24 Nitrogen absorption isotherm of (a) EDTA-0.0, (b) EDTA-1.0, (c) EDTA-2.5 and (d) EDTA-5.0.

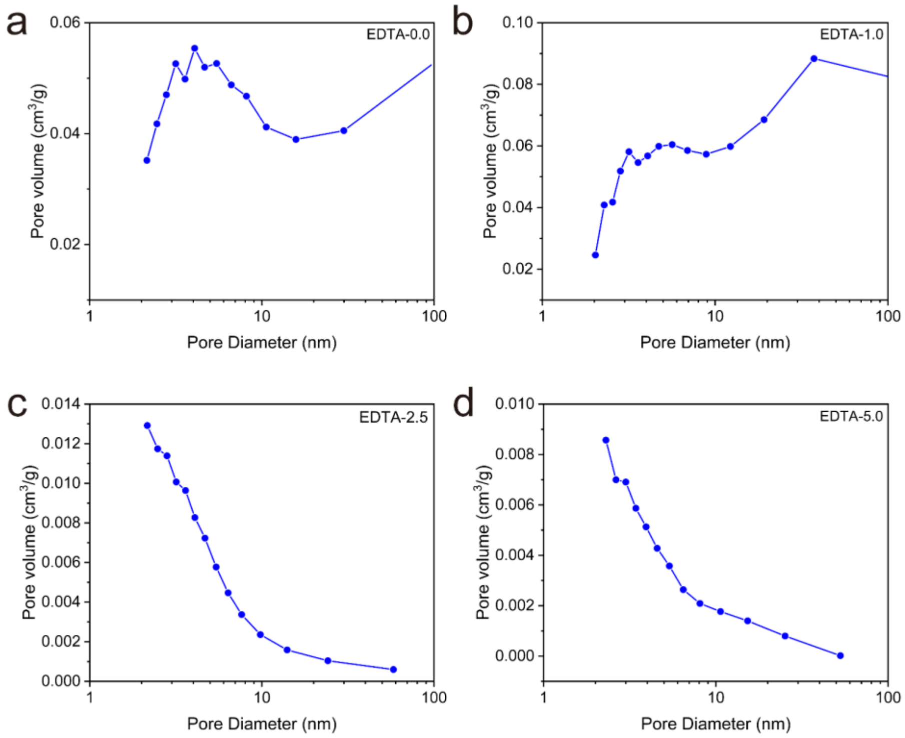

Figure. S25 Pore volume distribution of (a) EDTA-0.0, (b) EDTA-1.0, (c) EDTA-2.5 and (d) EDTA-5.0.

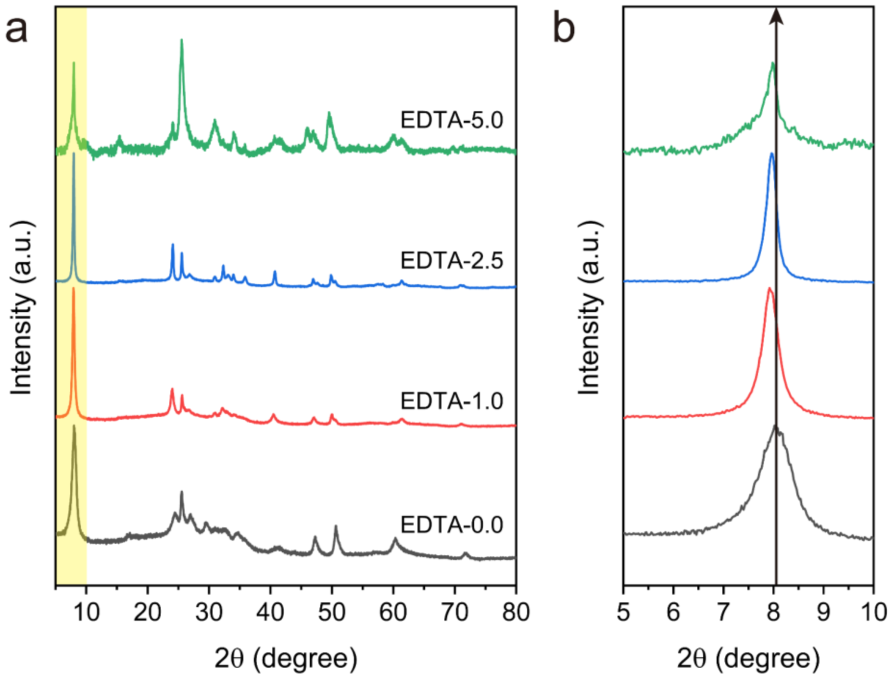

Figure. S26 (a) XRD patterns of EDTA-0.0, EDTA-1.0, EDTA-2.5 and EDTA-5.0. (b) The partially enlarged view of (001) plane at 5-10 °.

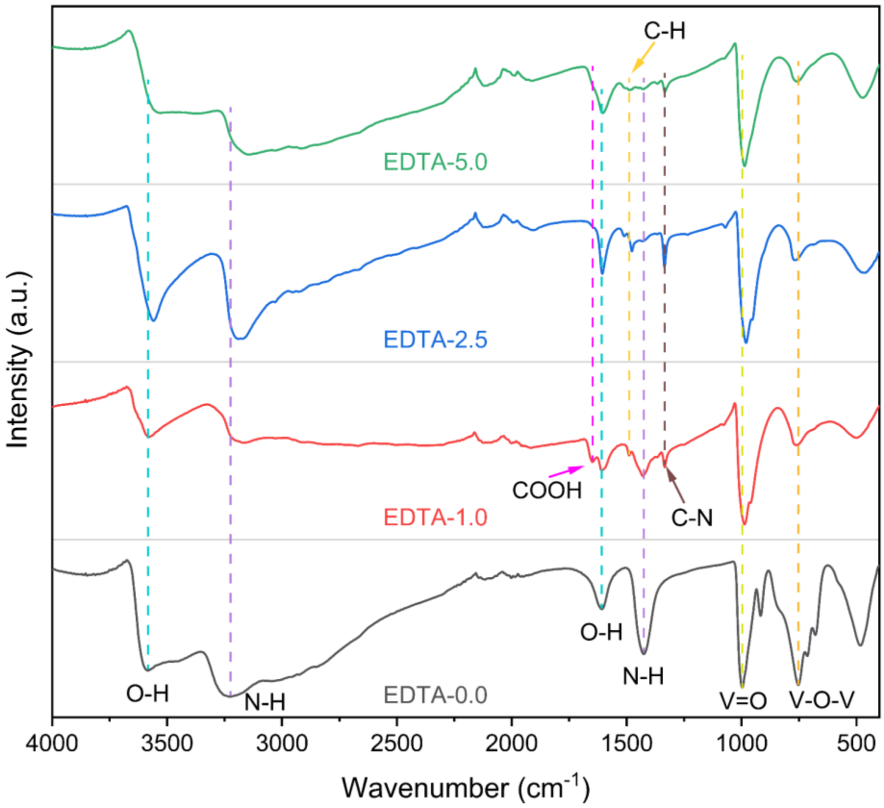

Figure. S27 FTIR spectra of EDTA-0.0, EDTA-1.0, EDTA-2.5 and EDTA-5.0.

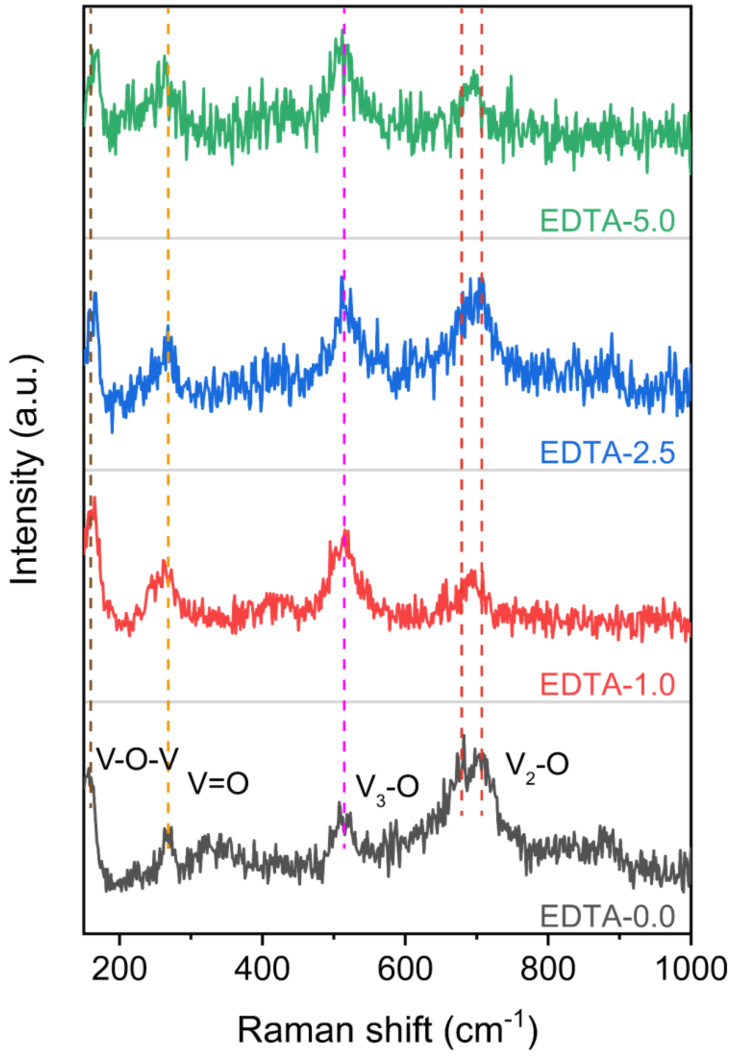

Figure. S28 Raman spectra of EDTA-0.0, EDTA-1.0, EDTA-2.5 and EDTA-5.0.

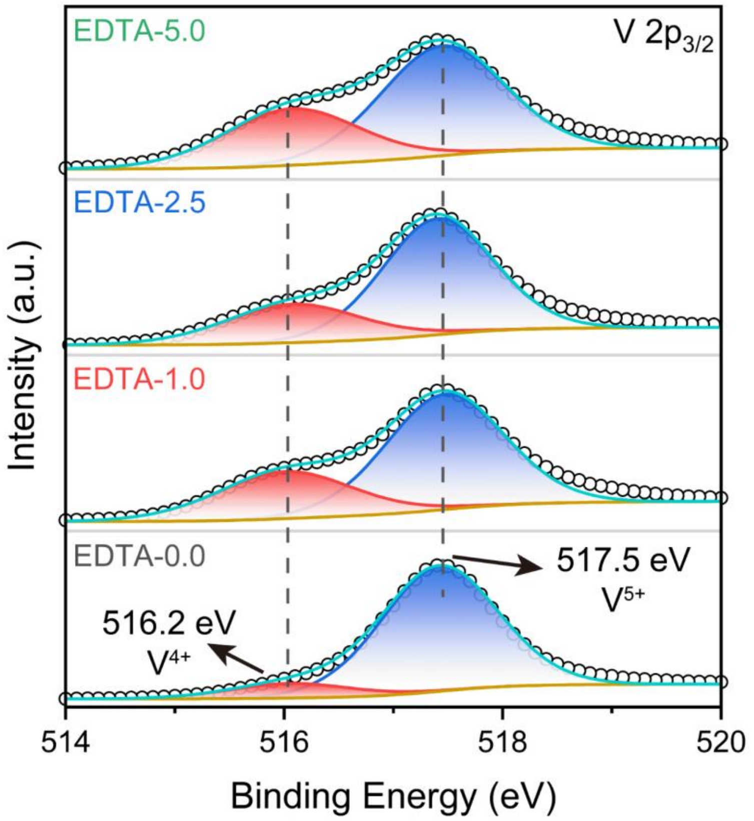

Figure. S29 The V 2p spectra of EDTA-0.0, EDTA-1.0, EDTA-2.5 and EDTA-5.0.

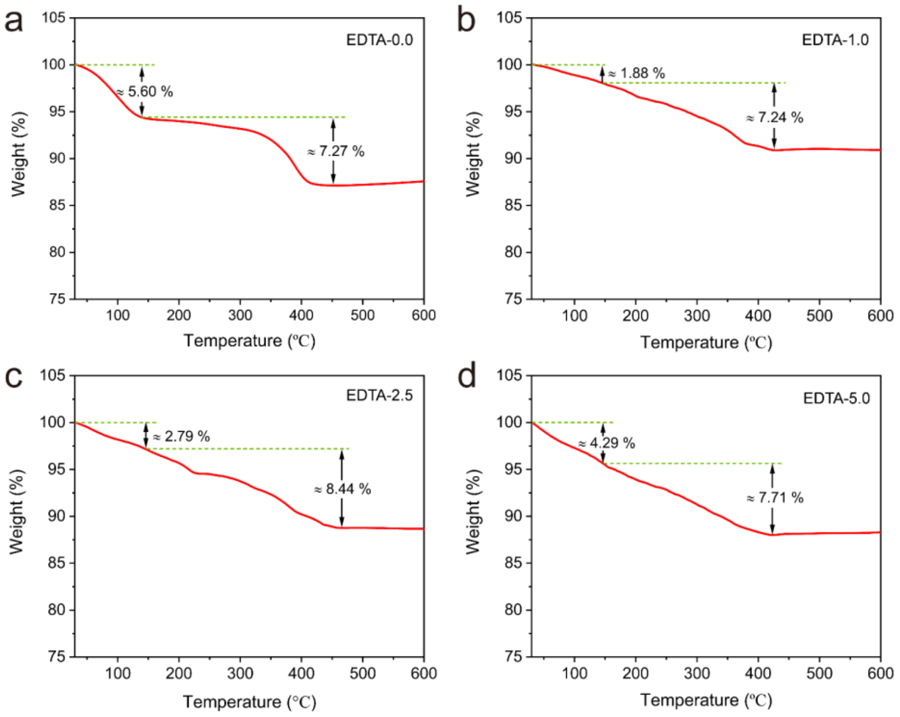

Figure. S30 The TGA curves of (a) EDTA-0.0, (b) EDTA-1.0, (c) EDTA-2.5 and (d) EDTA-5.0.

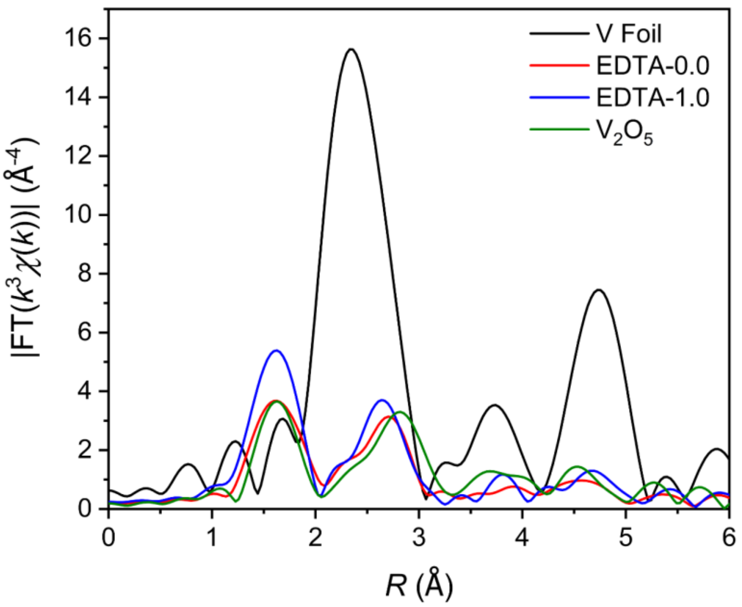

Figure. S31 The Fourier transform V K-edge EXAFS spectra of V foil, V_2O_5, EDTA-0.0 and EDTA-1.0 in *R* space.

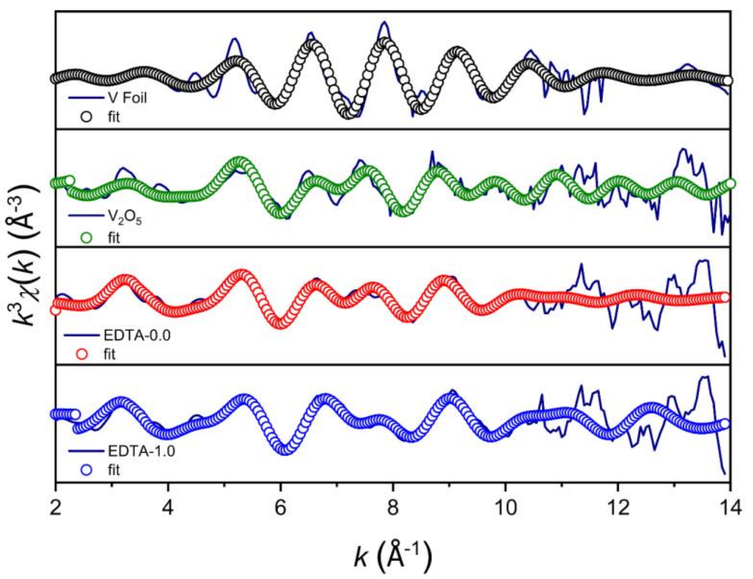

Figure. S32 Fitting curves of V foil, V_2O_5, EDTA-0.0 and EDTA-1.0 in *k* space.

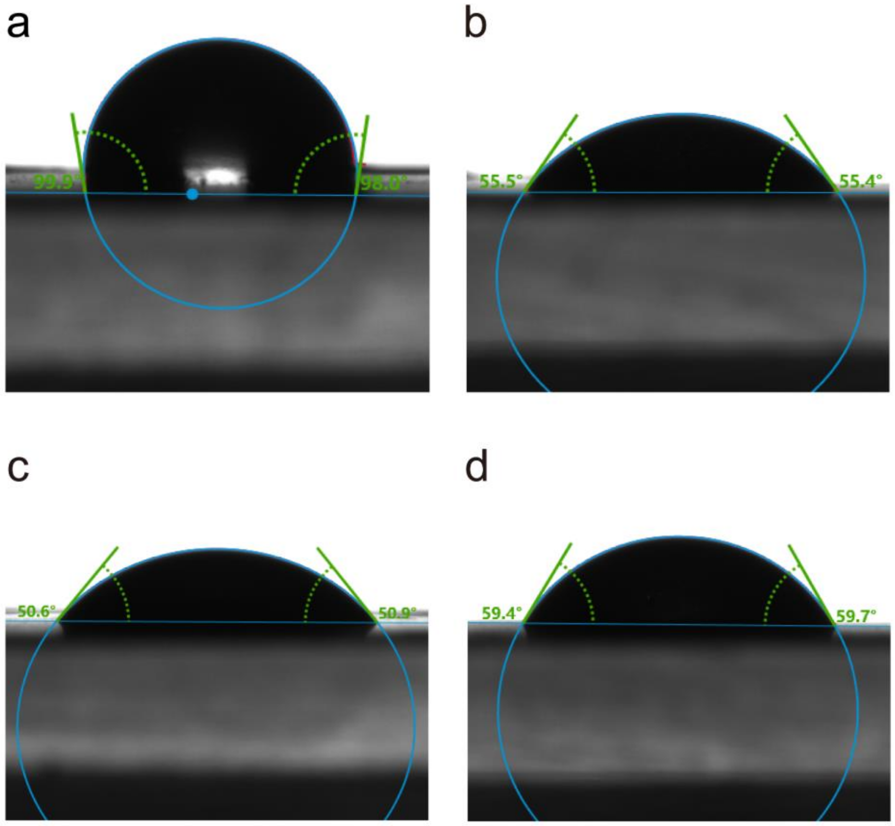

Figure. S33 The water contact angle of (a) EDTA-0.0, (b) EDTA-1.0, (c) EDTA-2.5 and (d) EDTA-5.0.

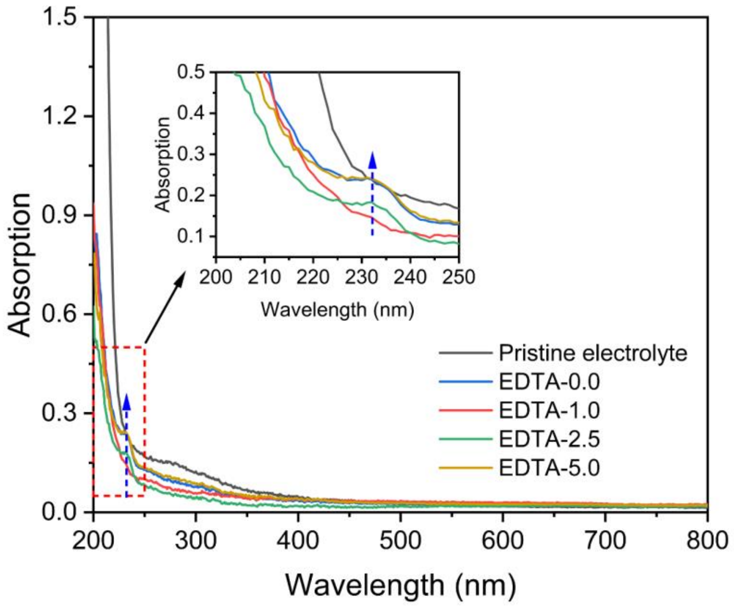

Figure. S34 UV-vis spectra of pristine electrolyte and electrolytes after electrodes soak.

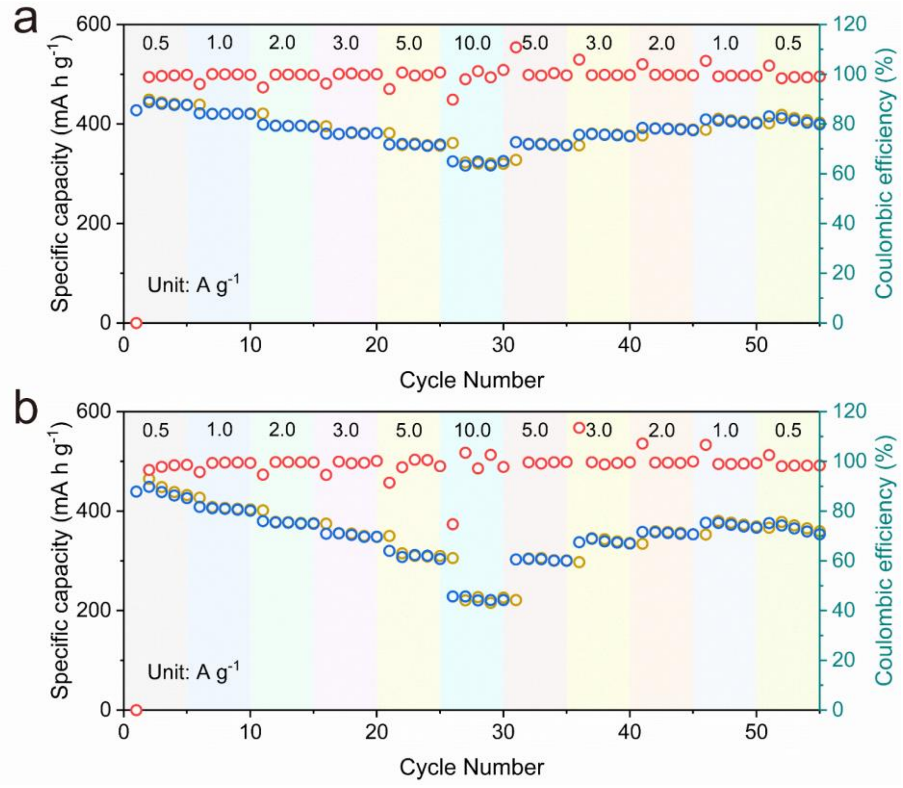

Figure. S35 Rate performance of (a) EDTA-2.5 and (b) EDTA-5.0 at the current density from 0.5 A g^{-1} to 10.0 A g^{-1}.

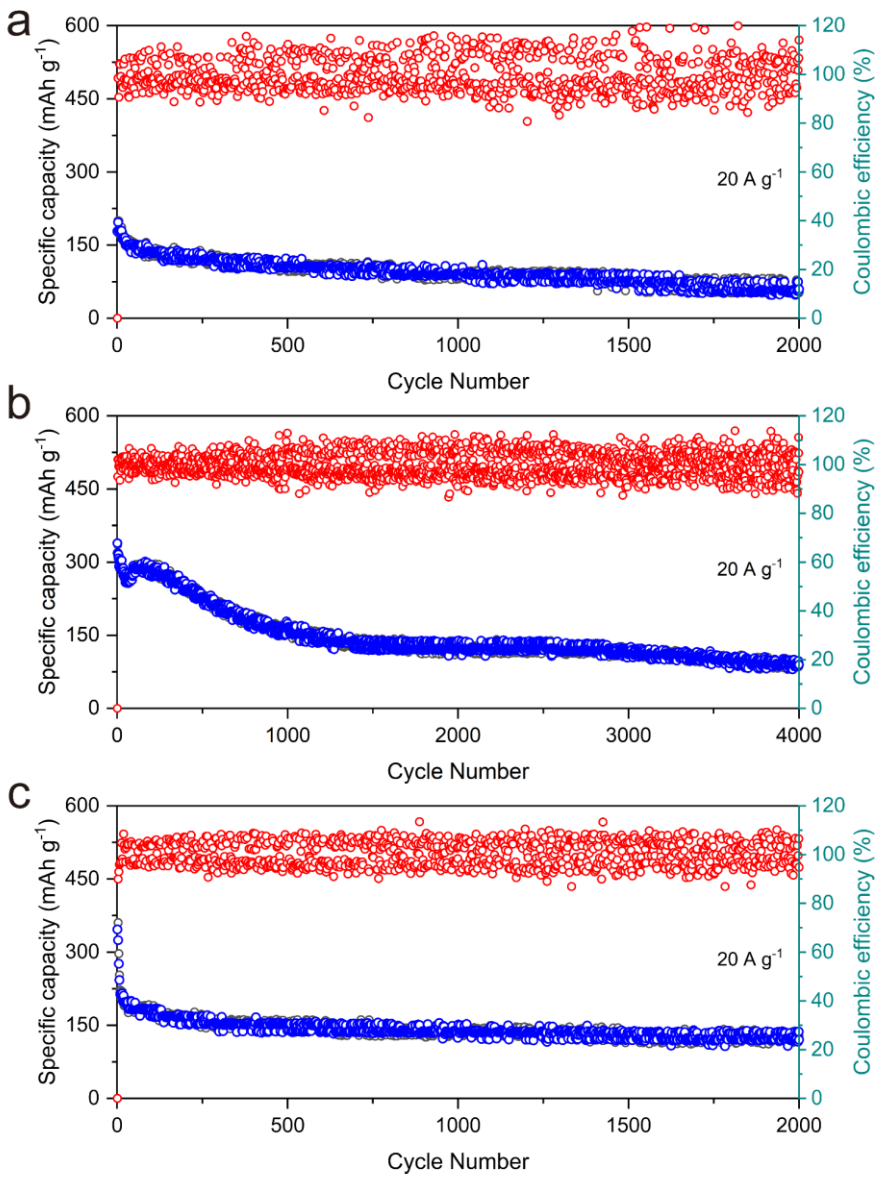

Figure. S36 The cycling performance of (a) EDTA-0.0, (b) EDTA-2.5 and (c) EDTA-5.0 cathode at the 20 A g^{-1}.

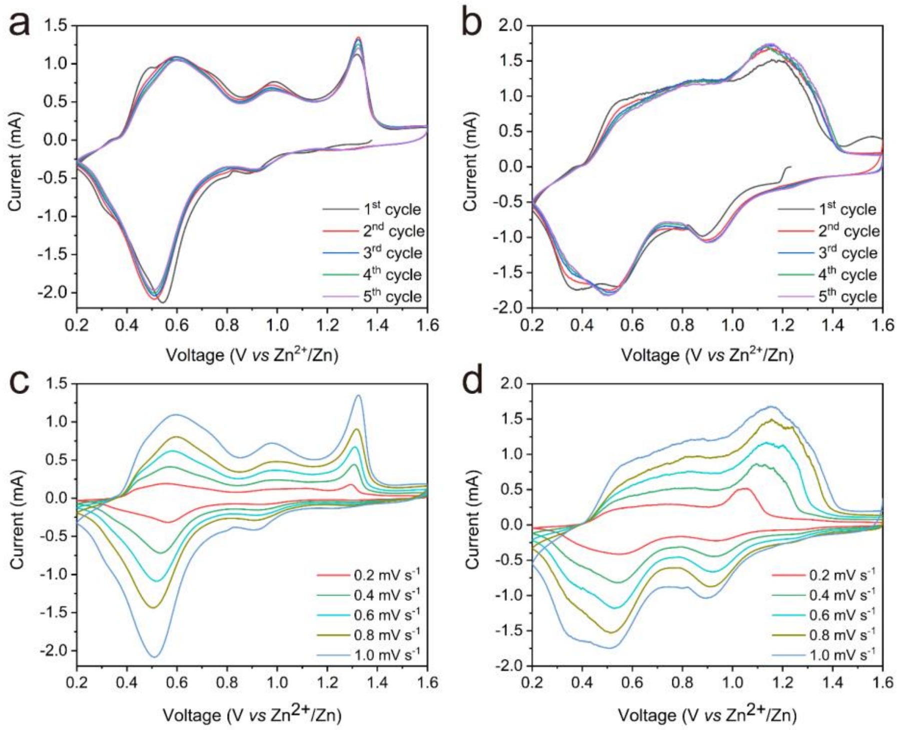

Figure. S37 The CV curves at 1.0 mV s^{-1} scan rate of (a) EDTA-2.5, and (b) EDTA-5.0. The CV curves at different scan rates of (c) EDTA-2.5, and (d) EDTA-5.0.

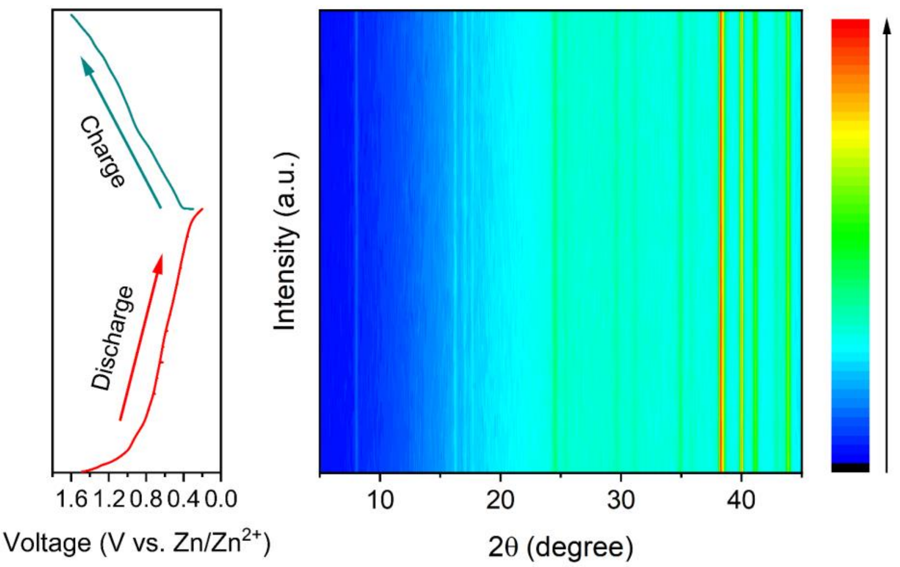

Figure. S38 In-suit XRD results of EDTA-1.0 cathode electrode.

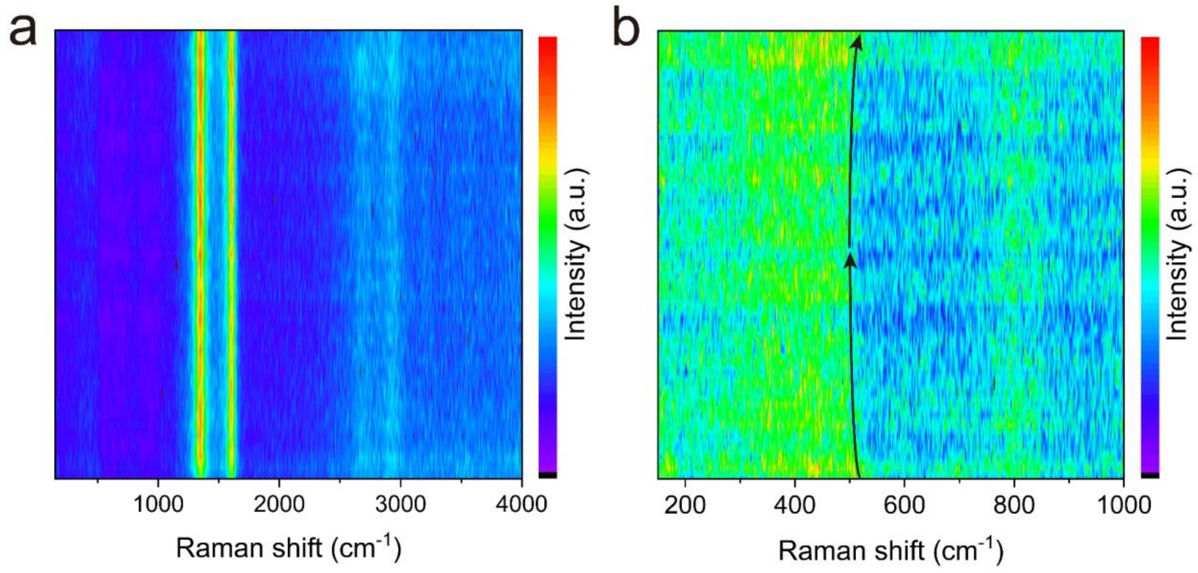

Figure. S39 In-suit Raman spectra of EDTA-1.0 cathode electrode.

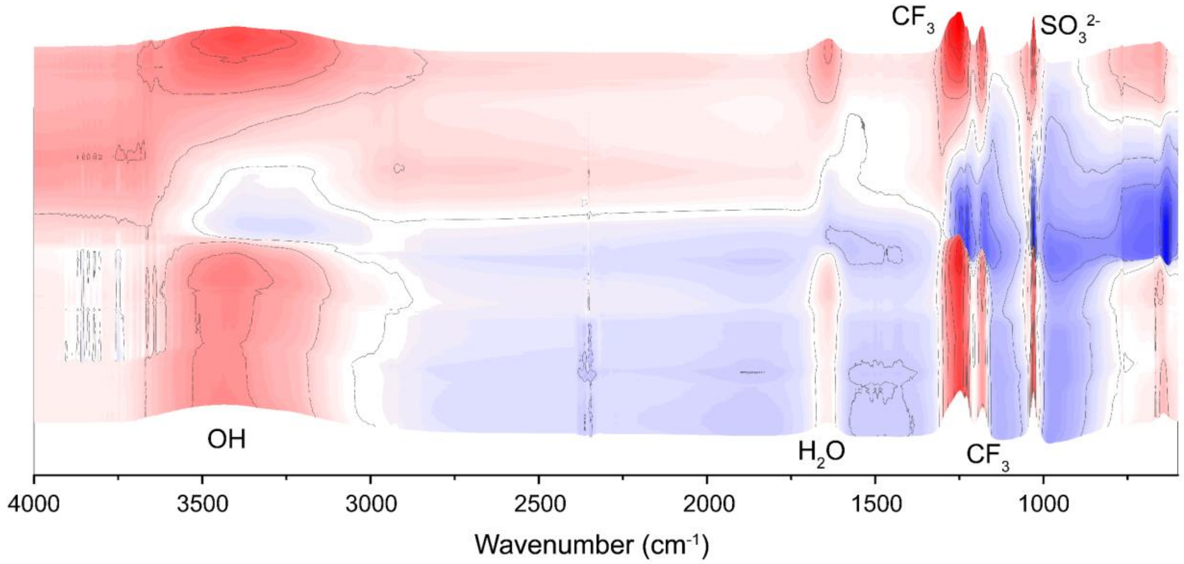

Figure. S40 In-suit FTIR spectra of EDTA-1.0 cathode.

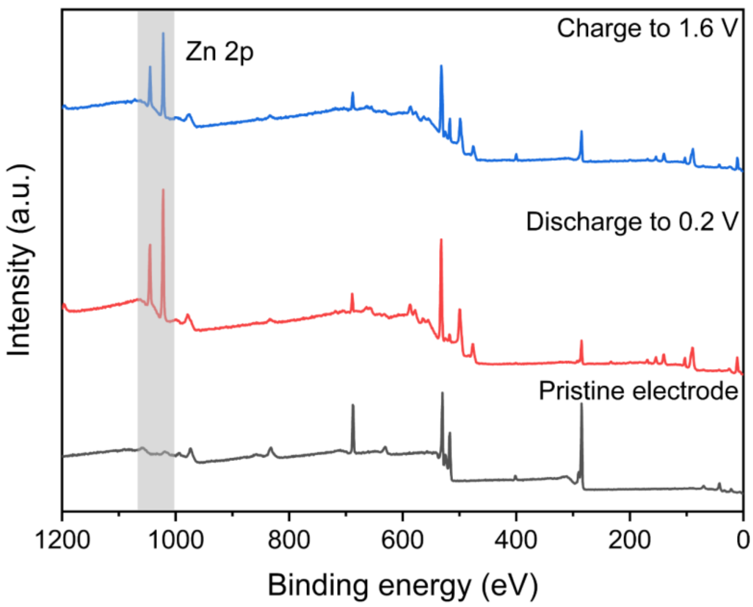

Figure. S41 The XPS full survey of pristine electrode, discharge to 0.2 V, and charge to 1.6 V.

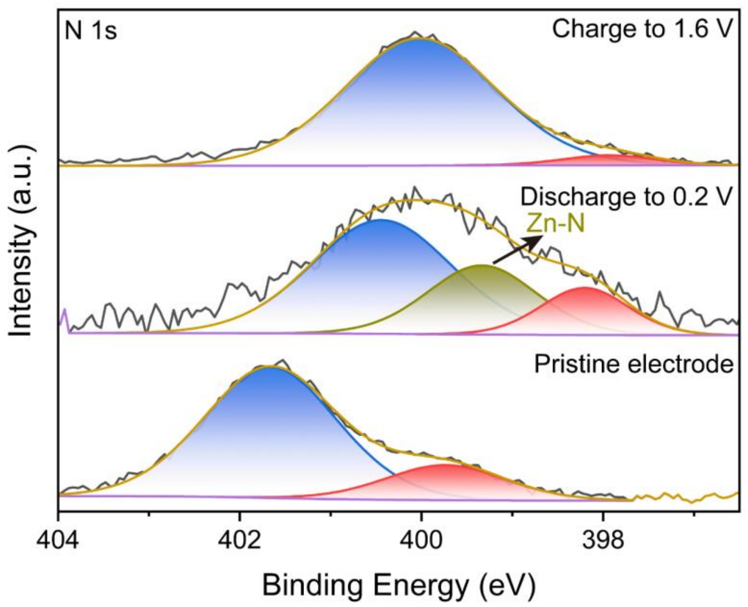

Figure. S42 High-resolution ex-situ XPS spectra of N 1s at the pristine, discharge to 0.2 V, and the following charging to 1.6 V states.

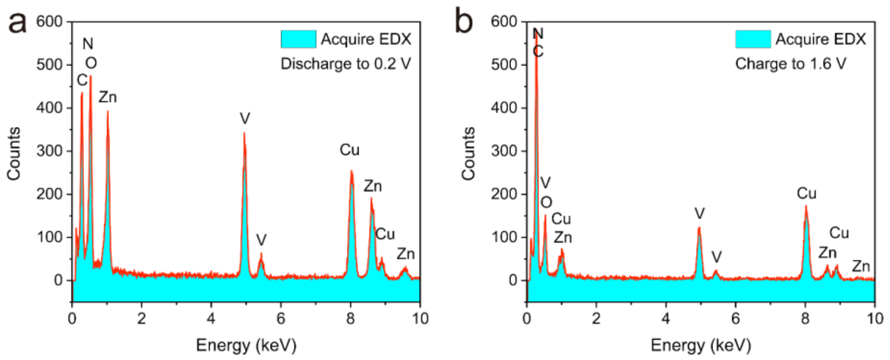

Figure. S43 EDX images of EDTA-1.0 at discharge to 0.2 V (a) and charge to 1.6 V (b).

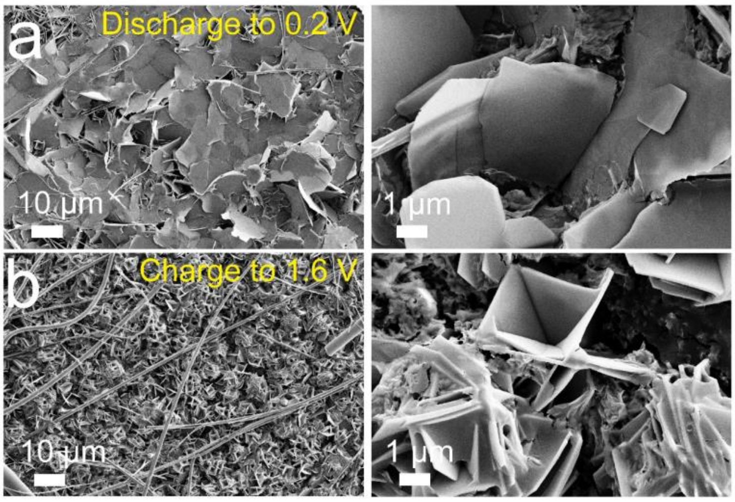

Figure. S44 Ex-situ SEM images of EDTA-1.0 at discharge to 0.2 V (a) and charge to 1.6 V (b).

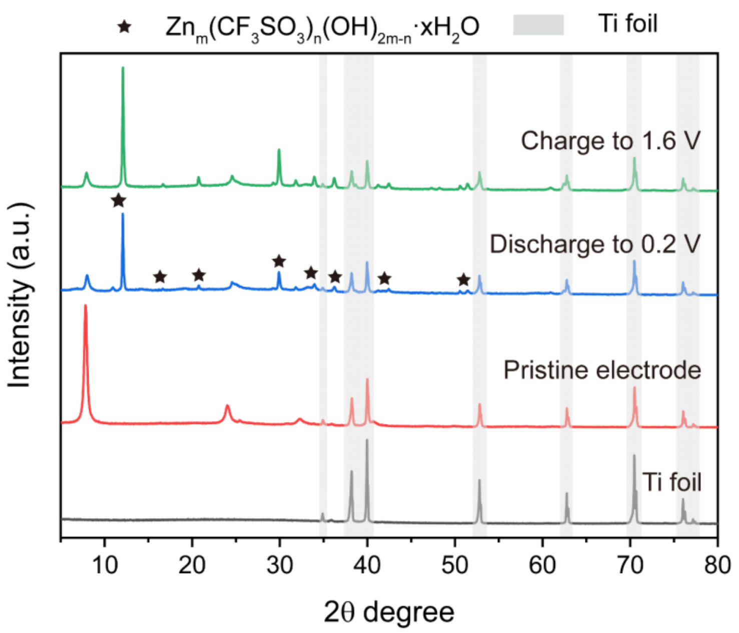

Figure. S45 The XRD patterns of Ti, pristine electrode, discharge to 0.2 V, and charge to 1.6 V.

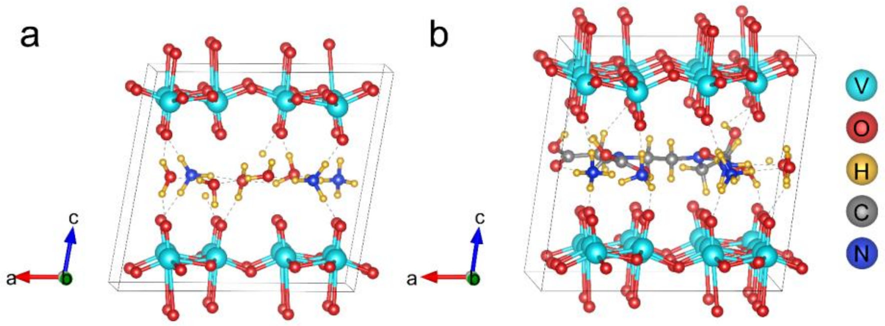

Figure. S46 Schematic diagram of the optimized structures of EDTA-0.0 (a) and EDTA-1.0 (b).

Table S1 C, H and N elemental analysis by CHNS test of None, Gly, DL-Ala, OA, MA, GA, OB, IDA, TA, CA, NTA and EDTA.

Samples	Weight (mg)	N (%)	C (%)	H (%)
None	1.271	2.216	0.223	1.520
Gly	1.256	2.255	1.880	1.281
DL-Ala	1.394	2.459	1.568	1.444
OA	1.264	2.701	2.551	1.404
MA	1.124	2.303	1.856	1.407
GA	1.362	2.372	1.823	1.486
OB	1.004	2.385	2.091	1.304
IDA	1.197	2.471	1.570	1.262
TA	1.160	2.348	1.147	1.341
CA	1.286	2.185	1.496	1.441
NTA	1.354	2.512	1.183	1.289
EDTA	1.163	2.346	1.672	1.305

Table S2 BET Specific Surface Area and Average Pore Diameter of None, Gly, DL-Ala, OA, MA, GA, OB, IDA, TA, CA, NTA and EDTA.

**Samples**	**BET Surface Area (m^2/g)**	**Average Pore Diameter (4V/A) (nm)**
None	24.051	14.416
Gly	63.099	9.392
DL-Ala	36.204	12.643
OA	58.326	9.614
MA	22.157	16.444
GA	29.871	14.669
OB	31.466	13.228
IDA	25.149	14.471
TA	21.749	17.034
CA	60.451	8.354
NTA	74.446	9.118
EDTA	27.005	19.124

Table S3 BET Specific Surface Area and Average Pore Diameter of EDTA-0.0, EDTA-1.0, EDTA-2.5 and EDTA-5.0.

**Samples**	**BET Surface Area (m^2/g)**	**Average Pore Diameter (4V/A) (nm)**
EDTA-0.0	24.051	14.416
EDTA-1.0	27.005	19.124
EDTA-2.5	30.950	16.225
EDTA-5.0	25.019	15.791

Table S4 C, H and N elemental analysis by CHNS test of EDTA-0.0, EDTA-1.0, EDTA-2.5 and EDTA-5.0.

Samples	Weight (mg)	N (%)	C (%)	H (%)
EDTA-0.0	1.271	2.216	0.223	1.520
EDTA-1.0	1.163	2.346	1.672	1.305
EDTA-2.5	1.271	2.127	0.757	1.255
EDTA-5.0	1.147	2.005	2.858	1.326

Table S5 EXAFS data fitting results of V foil, V_2O_5, EDTA-0.0 and EDTA-1.0.

Samples	Path	CN^a	$R(Å)^b$	σ^2 $(Å^2)^c$	$\Delta E_0(eV)^d$	*R* factor
			V K-edge (S_0^2=0.792)			
V foil	V-V	8*	2.635±0.029	0.0076	8.0	0.0043
	V-V	6*	3.013±0.019	0.0079		
V_2O_5	V-O	5.2±0.3	1.953±0.002	0.0014	4.6	0.0027
	V-O-V	6.8±0.9	3.226±0.010	0.0107	5.0	
EDTA-0.0	V-O	4.9±0.7	1.950±0.015	0.0019	5.1	0.0065
	V-O-V	5.7±0.5	3.202±0.011	0.0176	3.9	
EDTA-1.0	V-O	4.7±0.3	1.958±0.011	0.0012	1.2	0.0067
	V-O-V	6.1±0.7	3.016±0.018	0.0082	2.1	

[a]*CN*, coordination number; [b]*R*, the distance between absorber and backscatter atoms; [c]σ^2, the Debye Waller factor value; [d]ΔE_0, inner potential correction to account for the difference in the inner potential between the sample and the reference compound; *R* factor indicates the goodness of the fit. S_0^2 was fixed to 0.792, according to the experimental EXAFS fit of V foil by fixing *CN* as the known crystallographic value. * This value was fixed during EXAFS fitting, based on the known structure of V. Fitting conditions: *k* range：2.0-11.0; *R* range: 1.0-3.0; fitting space: R space; *k*-weight=3. A reasonable range of EXAFS fitting parameters: $0.800 < S_0^2 < 1.000$; $CN > 0$; $\sigma^2 > 0$ $Å^2$; $|\Delta E_0| < 10$ eV; *R* factor < 0.02.

Table S6 Comparison of the electrochemical properties of EDTA-1.0 with previously reported cathode materials.

Materials	Specific capacity		Capacity retention	References
δ-$Ni_{0.25}V_2O_5{\cdot}nH_2O$	402 mAh g^{-1} at 0.2 A g^{-1}	164 mAh g^{-1} at 5 A g^{-1}	98% after 1200 cycles at 5 A g^{-1}	[13]
$Zn_{0.25}V_2O_5{\cdot}H_2O$	282 mAh g^{-1} at 0.3 A g^{-1}	260 mAh g^{-1} at 2.4 A g^{-1}	80% after 1000 cycles at 2.4 A g^{-1}	[14]
$C_2H_8N_2V_7O_{16}$	382.6 mAh g^{-1} at 0.5 A g^{-1}	169.9 mAh g^{-1} at 10 A g^{-1}	65.3% after 10000 cycles at 5 A g^{-1}	[15]
HVO-MB	418 mAh g^{-1} at 0.05 A g^{-1}	243 mAh g^{-1} at 5 A g^{-1}	86.5% after 2000 cycles at 10 A g^{-1}	[16]
$Li_xV_2O_5{\cdot}nH_2O$	470 mAh g^{-1} at 0.5 A g^{-1}	170 mAh g^{-1} at 10 A g^{-1}	76% after 500 cycles at 5 A g^{-1}	[17]
$Ca_{0.67}V_8O_{20}{\cdot}3.5H_2O$/GN	466 mAh g^{-1} at 0.1 A g^{-1}	313 mAh g^{-1} at 2.4 A g^{-1}	74% after 2000 cycles at 5 A g^{-1}	[18]
KMgVOH	408 mAh g^{-1} at 0.1 A g^{-1}	210mAh g^{-1} at 2 A g^{-1}	72% after 2000 cycles at 4 A g^{-1}	[19]
VOH-PPy	422 mAh g^{-1} at 0.1 A g^{-1}	255 mAh g^{-1} at 2 A g^{-1}	54% after 5000 cycles at 10 A g^{-1}	[20]
LPVO	377 mAh g^{-1} at 0.1 A g^{-1}	195 mAh g^{-1} at 5 A g^{-1}	94% after 800 cycles at 5 A g^{-1}	[21]
EDTA-1.0	464.4 mAh g^{-1} at 0.5 A g^{-1}	324.4 mAh g^{-1} at 10 A g^{-1}	78% after 2000 cycle at 20 A g^{-1}	This work

Table S7 Comparison of Zn^{2+} diffusion coefficient for EDTA-1.0 and the previously reported cathodes in AZIBs.

**Materials**	**D_{Zn} (cm^2 s^{-1})**	**References**
$Zn_{0.25}V_2O_5 \cdot H_2O$	10^{-9} - 10^{-10}	[14]
Cu-$Bi_{2-x}Se_3$	10^{-10} -10^{-12}	[22]
C_8Q	$10^{-7.5}$ - $10^{-10.5}$	[23]
VS_2	10^{-9} - 10^{-11}	[24]
VO_2-D	10^{-7} - 10^{-10}	[25]
NVO-Rb	10^{-9} - 10^{-10}	[26]
PAF	10^{-9} - 10^{-11}	[27]
PEG-V_2O_5	10^{-9} - 10^{-11}	[28]
PANI-VOH	10^{-13} - 10^{-16}	[29]
EDTA-1.0	10^{-7} - 10^{-9}	This work

References:

1. He J, Zou Y, Huang Y *et al.* Interlayer ligand engineering of β-ni(oh)2 for oxygen evolution reaction. *Sci China Chem* 2020; **63**: 1684-93.
2. Zabinsky SI, Rehr JJ, Ankudinov A *et al.* Multiple-scattering calculations of x-ray-absorption spectra. *Physical Review B* 1995; **52**: 2995-3009.
3. Funke H, Chukalina M,Rossberg A. Wavelet analysis of extended x-ray absorption fine structure data. *Phys Scr* 2005; **2005**: 232.
4. Bin D, Huo W, Yuan Y *et al.* Organic-inorganic-induced polymer intercalation into layered composites for aqueous zinc-ion battery. *Chem* 2020; **6**: 968-84.
5. Frisch MJ, Trucks G, Schlegel HB *et al.* Gaussian 09 revision a.1. Gaussian inc. 2009.
6. Lu T,Chen F. Multiwfn: A multifunctional wavefunction analyzer. *J Comput Chem* 2012; **33**: 580-92.
7. Kresse G,Furthmüller J. Efficiency of ab-initio total energy calculations for metals and semiconductors using a plane-wave basis set. *Comput Mater Sci* 1996; **6**: 15-50.
8. Kresse G,Joubert D. From ultrasoft pseudopotentials to the projector augmented-wave method. *PHYSICAL REVIEW B* 1999; **59**: 1758-75.
9. Perdew JP, Burke K,Ernzerhof M. Generalized gradient approximation made simple. *Phys Rev Lett* 1996; **77**: 3865-8.
10. Dudarev SL, Botton GA, Savrasov SY *et al.* Electron-energy-loss spectra and the structural stability of nickel oxide: An lsda+u study. *Physical Review B* 1998; **57**: 1505-9.
11. Wang L, Maxisch T,Ceder G. Oxidation energies of transition metal oxides within the gga+u framework. *PHYSICAL REVIEW B* 2006; **73**.
12. Grimme S, Ehrlich S,Goerigk L. Effect of the damping function in dispersion corrected density functional theory. *J Comput Chem* 2011; **32**: 1456-65.
13. Li J, Mccoll K, Lu X *et al.* Multi-scale investigations of δ-ni0.25v2o5·nh2o cathode materials in aqueous zinc-ion batteries. *Adv Energy Mater* 2020; **10**: 2000058.
14. Kundu D, Adams BD, Duffort V *et al.* A high-capacity and long-life aqueous rechargeable zinc battery using a metal oxide intercalation cathode. *Nat Energy* 2016; **1**: 16119.
15. Ma X, Cao X, Yao M *et al.* Organic–inorganic hybrid cathode with dual energy-storage mechanism for ultrahigh-rate and ultralong-life aqueous zinc-ion batteries. *Adv Mater* 2022; **34**: 2105452.
16. Tong Y, Zang Y, Su S *et al.* Methylene blue intercalated vanadium oxide with synergistic energy storage mechanism for highly efficient aqueous zinc ion batteries. *J Energy Chem* 2023; **77**: 269-79.
17. Yang Y, Tang Y, Fang G *et al.* Li+ intercalated v2o5·nh2o with enlarged layer spacing and fast ion diffusion as an aqueous zinc-ion battery cathode. *Energy Environ Sci* 2018; **11**: 3157-62.
18. Zhu K, Wu T,Huang K. A high capacity bilayer cathode for aqueous zn-ion batteries. *ACS Nano* 2019; **13**: 14447-58.
19. Feng Z, Zhang Y, Sun J *et al.* Dual ions enable vanadium oxide hydration with superior zn2+ storage for aqueous zinc-ion batteries. *Chem Eng J* 2022; **433**: 133795.
20. Feng Z, Sun J, Liu Y *et al.* Engineering interlayer space of vanadium oxide by pyridinesulfonic acid-assisted intercalation of polypyrrole enables enhanced aqueous zinc-ion storage. *ACS Appl Mater Interfaces* 2021; **13**: 61154-65.
21. He W, Fan Z, Huang Z *et al.* A li+ and pani co-intercalation strategy for hydrated v2o5 to enhance zinc ion storage performance. *J Mater Chem A* 2022; **10**: 18962-71.
22. Zong Y, Chen H, Wang J *et al.* Cation defect-engineered boost fast kinetics of two-dimensional topological bi2se3 cathode for high-performance aqueous zn-ion batteries. *Adv Mater* 2023; **35**: 2306269.

23. Ding C, Wang Y, Li C *et al.* Constructing ultra-stable, high-energy, and flexible aqueous zinc-ion batteries using environment-friendly organic cathodes. *Chem Sci* 2024; **15**: 4952-9.
24. Pan R, Cui F, Zheng A *et al.* Achieving synergetic anion-cation redox chemistry in freestanding amorphous vanadium oxysulfide cathodes toward ultrafast and stable aqueous zinc-ion batteries. *Adv Funct Mater* 2023; **33**: 2300619.
25. He Q, Hu T, Wu Q *et al.* Tunnel-oriented vo2 (b) cathode for high-rate aqueous zinc-ion batteries. *Adv Mater* 2024; **36**: 2400888.
26. Wang K, Li S, Chen X *et al.* Trifunctional rb+-intercalation enhancing the electrochemical cyclability of ammonium vanadate cathode for aqueous zinc ion batteries. *ACS Nano* 2024; **18**: 7311-23.
27. Gong S, Chao Y, Yang F *et al.* Bifunctional potential structure design breaks electrolyte limitations of zinc ion battery. *Angew Chem Int Ed* 2024; **63**: e202401629.
28. Lin C, Qi F, Dong H *et al.* Suppressing vanadium dissolution of v2o5via in situ polyethylene glycol intercalation towards ultralong lifetime room/low-temperature zinc-ion batteries. *Nanoscale* 2021; **13**: 17040-8.
29. Wang M, Zhang J, Zhang L *et al.* Graphene-like vanadium oxygen hydrate (voh) nanosheets intercalated and exfoliated by polyaniline (pani) for aqueous zinc-ion batteries (zibs). *ACS Appl Mater Interfaces* 2020; **12**: 31564-74.
